# Polygenic risk-stratified screening for nasopharyngeal carcinoma in high-risk endemic areas of China: a cost-effectiveness study

**DOI:** 10.3389/fpubh.2024.1375533

**Published:** 2024-05-02

**Authors:** Da-Wei Yang, Jacob A. Miller, Wen-Qiong Xue, Minzhong Tang, Lin Lei, Yuming Zheng, Hua Diao, Tong-Min Wang, Ying Liao, Yan-Xia Wu, Xiao-Hui Zheng, Ting Zhou, Xi-Zhao Li, Pei-Fen Zhang, Xue-Yin Chen, Xia Yu, Fugui Li, Mingfang Ji, Ying Sun, Yong-Qiao He, Wei-Hua Jia

**Affiliations:** ^1^School of Public Health, Sun Yat-Sen University, Guangzhou, China; ^2^State Key Laboratory of Oncology in South China, Collaborative Innovation Center for Cancer Medicine, Guangdong Key Laboratory of Nasopharyngeal Carcinoma Diagnosis and Therapy, Sun Yat-sen University Cancer Center, Guangzhou, China; ^3^Department of Radiation Oncology, Cleveland Clinic, Cleveland, OH, United States; ^4^Wuzhou Red Cross Hospital, Wuzhou, Guangxi, China; ^5^Shenzhen Center for Chronic Disease Control, Shenzhen, China; ^6^Cancer Research Institute of Zhongshan City, Zhongshan Hospital of Sun Yat-sen University, Zhongshan, China; ^7^Guangdong Key Laboratory of Nasopharyngeal Carcinoma Diagnosis and Therapy, State Key Laboratory of Oncology in South China, Collaborative Innovation Center for Cancer Medicine, Department of Radiation Oncology, Sun Yat-sen University Cancer Center, Guangzhou, China

**Keywords:** polygenic risk stratification, nasopharyngeal carcinoma, screening, cost-effectiveness, modeling study

## Abstract

**Background:**

Nasopharyngeal carcinoma (NPC) has an extremely high incidence rate in Southern China, resulting in a severe disease burden for the local population. Current EBV serologic screening is limited by false positives, and there is opportunity to integrate polygenic risk scores for personalized screening which may enhance cost-effectiveness and resource utilization.

**Methods:**

A Markov model was developed based on epidemiological and genetic data specific to endemic areas of China, and further compared polygenic risk-stratified screening [subjects with a 10-year absolute risk (AR) greater than a threshold risk underwent EBV serological screening] to age-based screening (EBV serological screening for all subjects). For each initial screening age (30–34, 35–39, 40–44, 45–49, 50–54, 55–59, 60–64, and 65–69 years), a modeled cohort of 100,000 participants was screened until age 69, and then followed until age 79.

**Results:**

Among subjects aged 30 to 54 years, polygenic risk-stratified screening strategies were more cost-effective than age-based screening strategies, and almost comprised the cost-effectiveness efficiency frontier. For men, screening strategies with a 1-year frequency and a 10-year absolute risk (AR) threshold of 0.7% or higher were cost-effective, with an incremental cost-effectiveness ratio (ICER) below the willingness to pay (¥203,810, twice the local *per capita* GDP). Specifically, the strategies with a 10-year AR threshold of 0.7% or 0.8% are the most cost-effective strategies, with an ICER ranging from ¥159,752 to ¥201,738 compared to lower-cost non-dominated strategies on the cost-effectiveness frontiers. The optimal strategies have a higher probability (29.4–35.8%) of being cost-effective compared to other strategies on the frontier. Additionally, they reduce the need for nasopharyngoscopies by 5.1–27.7% compared to optimal age-based strategies. Likewise, for women aged 30–54 years, the optimal strategy with a 0.3% threshold showed similar results. Among subjects aged 55 to 69 years, age-based screening strategies were more cost-effective for men, while no screening may be preferred for women.

**Conclusion:**

Our economic evaluation found that the polygenic risk-stratified screening could improve the cost-effectiveness among individuals aged 30–54, providing valuable guidance for NPC prevention and control policies in endemic areas of China.

## Introduction

Nasopharyngeal carcinoma (NPC) exhibits a distinctive ethnic and geographical distribution, with high incidence rates in Southern China, Southeast Asia and the Middle East/North Africa (1–4). According to World Health Organization estimates, China accounted for nearly half of the global NPC burden in 2020 (2–4). Currently, the Chinese Ministry of Health recommends using anti-EBV IgA (VCA-IgA and EBNA1-IgA) serological tests for NPC screening in individuals aged 30 to 69 years in NPC endemic areas (5). While this age-based screening strategy has shown improvements in early diagnosis rate from 22.4 to 79.0%, and may reduce NPC mortality, it is limited by low positive predictive value (PPV) of only about 4%, meaning that more than 95% of individuals who test positive do not have NPC (6–8). This leads to excessive laboratory tests, endoscopic examinations, and imaging, thereby increasing both screening costs and medical visits (8, 9). Consequently, there is a need to improve the performance of NPC screening by reducing unnecessary clinical examinations while maintaining cost-effectiveness.

Recent large-scale population studies have highlighted the potential of using a polygenic risk score (PRS), which incorporates the effects of SNPs (Single Nucleotide Polymorphism), to identify individuals at high risk of cancer (10–13). This approach has emerged as a promising tool for personalized screening in breast cancers and prostate cancers, and has also demonstrated to improve the cost-effectiveness and the benefit-to-harm ratio (14–20). Given the regional distribution disparities, familial clustering, and high heritability of NPC, genetic factors are believed to play an important role in its etiology (4, 21–25). This makes NPC an ideal candidate for developing a PRS for risk stratification, especially in high-risk regions of Southern China. Our previous study conducted a multi-center large-scale genome-wide association study of NPC and developed a PRS comprising 12 SNPs (26). By using this PRS to select high-risk individuals for EBV serological screening, we observed a substantial improvement in PPV, particularly in the top 20 and 5% of the PRS, with the PPV reaching 7.99 and 11.91%, respectively (26).

With the declining costs of sequencing, the detection cost of PRS has become more affordable, indicating its potential economic viability for large-scale screening. As highlighted by the 2021 Polygenic Risk Score Task Force of the International Common Disease Alliance, the lack of economic evidence regarding the large-scale screening application of PRS underscores an urgent need for research (13). Therefore, further evidence and evaluation are required for the implementation of PRS into NPC screening programs and its cost-effectiveness. It is essential to assess how a polygenic risk-stratified approach can be integrated into existing EBV serological screening strategies, including considerations such as the selection of high-risk individuals for screening and determining the appropriate screening frequency.

In light of this, we conducted a comprehensive cost-effectiveness analysis to compare the age-based screening strategy with the polygenic risk-stratified screening strategy. By evaluating and comparing the cost-effectiveness of these two screening strategies with different risk thresholds and screening frequencies, we aim to provide valuable insights for decision-making and resource allocation in NPC screening.

## Materials and methods

### Markov decision-analytic model

A natural history model was constructed to simulate the NPC progression and calculate the related health and economic outcomes in the Chinese population in high-risk endemic areas (Supplementary Figure S1), and we further calibrated and validated this natural history model in detail (Supplementary Methods). The Markov model consisted of 15 health states, encompassing perfect health, five separate stages of undetected NPC (preclinical stage I, II, III, IVA/B, IVC), five separate stages of detected NPC (clinical stage I, II, III, IVA/B, IVC), three separate prognostic states (local recurrence, regional recurrence, and distant metastasis), and death. And we considered different initial screening age groups, specifically 30–34, 35–39, 40–44, 45–49, 50–54, 55–59, 60–64, and 65–69. Each age group consists of a hypothetical cohort of 100,000 participants who were followed up until the age of 79 years or death (27). The overall results of each age group were calculated assuming the uniform distribution of ages within each age group. The model adopted a cycle length of 3 months, and a half-cycle correction was applied. Further details about the model were described in Supplementary Methods, and its parameters were shown in Supplementary Table S1. The study followed the Consolidated Health Economic Evaluation Reporting Standards (CHEERS) reporting guideline was approved by the ethics committee of the Sun Yat-sen University Cancer Center. The study data were deposited at the Research Data Deposit platform (RDDA2024193982, https://www.researchdata.org.cn)

### Evaluated screening strategies

Three screening strategies were simulated using the Markov model: no screening, age-based screening, and polygenic risk-stratified screening strategies (Supplementary Figure S2). In the age-based screening strategy, individuals with a specific initial age underwent EBV serological (VCA IgA and EBNA IgA) screening every 1–5 years (abbreviated as Age-1 to Age-5). In the polygenic risk-stratified screening, all individuals were required to undergo PRS testing only once at the onset of the screening. They were further identified as high-risk individuals based on their 10-year absolute risk (AR) values, which were estimated using their PRS and the designated screening age. These high-risk individuals then underwent EBV serology tests every 1–5 years. The thresholds for 10-year absolute risk varied for men (ranging from 0.3 to 1.0%) and for women (ranging from 0.1 to 0.3%), considering the proportion of high-risk individuals in the general population. When an individual’s 10-year absolute risk surpassed the threshold, they would begin the subsequent EBV serological tests. Screening continued until either the individual’s fell below the threshold or they reached the age of 69. The polygenic risk-stratified strategies are labeled as AR-10 years of NPC absolute risk threshold-screening frequency.

### Polygenic risk profiles

In our previous study, we conducted a large-scale multi-center genome-wide association study on NPC, which involved 4,506 NPC patients and 5,384 controls (26). The objective was to identify SNPs associated with NPC and construct a PRS model for identifying individuals at high risk of developing the disease. Using the 12 identified SNPs, we constructed a PRS model. For the current study, we randomly selected 1,118 individuals from the Guangdong Biobank Cohort Study (ChiCTR1800015736) (28), which represent the general population residing in high-risk endemic areas of NPC (8). We detected the 12 SNPs and applied the PRS model to calculate the distribution of PRS. And we calculated gender- and age-specific 10-year AR values for the population by using age and PRS (26). Further details on how the Markov model was constructed based on polygenic risk profiles can be found in Supplementary Table S2 and Supplementary Figures S3, S4.

### Transition probabilities

The transition probabilities among undetected stages of NPC and the stage-specific probability of symptomatic manifestation (undetected to detected NPC) were estimated and calibrated by using incidence rate of NPC from Guangzhou registry data of CI5 (1). Additionally, the transitions probabilities from diagnosis to relapse/metastasis and death were estimated base on real-world data from the big-data platform of Sun Yat-sen University Cancer Center (29). The detailed descriptions how to estimate, calibrate, and validate the transition probabilities can be found in Supplementary Figures S5–S8 and Supplementary Tables S3–S7.

### Health state utilities and costs

The utilities of each state in Markov model were derived from a previous study, which defined time-dependent health utilities for NPC patients (30). The detailed values for the utilities were shown in Supplementary Table S1. The cost of each screening strategy comprises three main components: screening, diagnosis and treatment of NPC patients, and follow-up care for patients. The main screening cost included the cost of testing two anti-EBV IgA antibodies, amounting to ¥108.61 referred to previous study, and the cost of PRS, which was ¥120.00 after considering the reagents of testing 12 SNPs, wastage, estimation of polygenic risk, and others. The detailed costs for each component were described in Supplementary Table S8. All costs were reported Chinese renminbi (RMB, ¥) (to convert to US$, divided by 6.7). We assumed a standard discount rate of 3% for both utilities (quality-adjusted life-years, QALYs) and costs, in accordance with previous studies (27, 30–34).

### Outcome measures

The primary outcome measures included: (1) the cost-effectiveness efficiency frontier: this refers to the line segments that connect a set of strategies that achieve the maximum health benefit relative to their cost at a given level. The efficiency frontier helps compare the health outcomes of different screening strategies and identifies the most efficient strategies within a given budget constraint. The others strategies not on the efficiency frontier were considered to be dominated strategies. A strongly dominated strategy was defined as a strategy for which there exists another strategy that yielded better health benefit at a lower cost. A weakly dominated strategy was defined as a strategy that is dominated by a linear combination of two other strategies. (2) The incremental cost-effectiveness ratios (ICER), defined as the incremental cost divided by incremental QALYs (32, 35).

The cost-effective screening strategies were identified on the following criteria: (1) were located on the cost-effectiveness efficiency frontier. Those strategies were considered to be the most efficient options at different cost level, providing the best balance between cost and effectiveness. (2) had an ICER less than the willingness to pay (WTP) threshold compared with the preceding strategy on the efficiency frontier. If the ICER was below the WTP threshold, the strategy was considered cost-effective. Recognizing limitations in WTP thresholds, we utilized a threshold of twice the local *per capita* GDP based on WHO-CHOICE guidelines (31). For example, the per-capita GDP of Guangdong province in 2022 was ¥101,905, the WTP threshold would be ¥203,810, representing the economic level of Southern China (6, 31).

Secondary outcomes included reduction in NPC mortality, incremental QALYs gained via screening, resource utilization (serology tests, nasopharyngoscopies), and early-stage NPC case detection rates. All outcomes are reported per 100,000 subjects from the general population.

### Sensitivity analysis

To assess the robustness and variability of the results, sensitivity analyses were further conducted for each age group (16, 36). Probabilistic sensitivity analysis was conducted through 1,000 simulations to evaluate the probability of each screening strategy on the efficiency frontier being cost-effective relative to the others on the same frontier. Univariate sensitivity analysis was performed for all parameters within their respective ranges to identify the most sensitive parameters. The optimal strategy of each age group underwent univariate sensitivity analysis. To facilitate observation of the changes in ICERs, the resulting values were reported as the ratio of ICERs after variable changes to the ICERs obtained in base case analysis. In addition, compliance with screening strategies was also considered in univariate sensitivity analysis owing to its large variability in different regions. We performed all statistical analyses using R software, version 4.0.3 (R Foundation for Statistical Computing).

## Results

### Base-case analysis for age-based screening strategies

We first evaluated the cost-effectiveness of age-based screening strategies and further explored the optimal screening frequency of age-based screening strategies. In this base-case analysis, the strategies on the cost-effectiveness efficiency frontiers included those strategies with screening frequencies from 5 to 1 year (Age-5, Age-4, Age-3, Age-2, and Age-1). As shown in Table 1 and Supplementary Figure S9, for men aged 30–59 (30–34, 35–39, 40–44, 45–49, 50–54, and 55–59 groups), the strategies of screening every 3–5 years are likely to be cost-effective (ICER less than WTP that was twice the *per capita* GDP), and a strategy with a 3-year frequency (Age-3) was most cost-effective, with an ICER between ¥122,976 and ¥186,489. For men aged 60–64, the strategies of screening every 4–5 years were cost-effective. And the strategy of screening every 4 years (Age-4) was most cost-effective, with an ICER of ¥180,529. For men aged 65–69, the one-time screening strategy was considered to be the most cost-effective strategy with an ICER of ¥124,865. For women aged 30–54 (include 30–34, 35–39, 40–44, 45–49 and 50–54 groups), the strategy of screening every 5 years was found to be the cost-effective strategy, with an ICER ranging from ¥165,186 to ¥203,150. However, for women aged 55 and above, age-based screening strategies were not considered cost-effective based on the predefined willingness-to-pay threshold (Supplementary Table S9; Supplementary Figure S10).

**Table 1:** Base-case analysis of only age-based screening strategies in male population.

Starting age	Screening strategy^a^	Incremental costs^b^	Incremental QALYs^b^	ICER^c^	
				vs. no screening	vs. preceding strategy on efficiency frontier
30–34	Age-5	52,263,435	840	62,253	62,253
	Age-4	63,477,407	962	65,967	91,366
	Age-3	82,980,500	1,113	74,527	129,014
	Age-2	122,160,233	1,298	94,119	212,356
	Age-1	243,353,757	1,536	158,397	508,329
35–39	Age-5	48,042,990	851	56,476	56,476
	Age-4	58,341,768	967	60,306	88,214
	Age-3	76,244,383	1,112	68,583	124,089
	Age-2	112,015,315	1,287	87,038	204,089
	Age-1	222,440,579	1,513	146,990	487,898
40–44	Age-5	43,688,206	763	57,228	57,228
	Age-4	53,055,184	868	61,148	89,854
	Age-3	68,403,058	992	68,923	122,976
	Age-2	100,503,389	1,148	87,528	206,061
	Age-1	199,699,464	1,350	147,916	491,434
45–49	Age-5	38,728,257	685	56,505	56,505
	Age-4	47,168,526	770	61,227	99,300
	Age-3	60,427,104	871	69,374	131,746
	Age-2	88,539,952	998	88,684	220,765
	Age-1	174,302,204	1,163	149,916	521,991
50–54	Age-5	33,477,743	511	65,519	65,519
	Age-4	39,767,340	569	69,905	108,603
	Age-3	51,960,292	646	80,381	157,226
	Age-2	74,447,473	736	101,132	250,641
	Age-1	146,080,468	858	170,207	586,645
55–59	Age-5	27,382,976	365	75,055	75,055
	Age-4	32,351,879	401	80,693	137,697
	Age-3	40,928,040	447	91,579	186,489
	Age-2	59,552,813	508	117,149	303,160
	Age-1	114,013,919	586	194,440	698,041
60–64	Age-5	20,413,038	202	101,070	101,070
	Age-4	23,935,730	221	108,071	180,529
	Age-3	29,513,900	244	120,741	242,984
	Age-2	40,875,553	275	148,604	371,001
	Age-1	77,052,913	320	240,950	808,898
65–69	Age-5	11,836,613	95	124,865	124,865
	Age-4	13,777,888	103	133,174	224,113
	Age-3	15,846,782	111	142,886	277,805
	Age-2	19,979,588	121	164,705	397,379
	Age-1	32,600,377	137	237,569	792,777

^a^The screening strategies are labeled as follows: for age-based strategies, “Age-screening frequency.” For example, “Age-3” indicated that all individuals undergo an EBV serological test every 3 years.^b^Incremental health benefits gained, and costs incurred from age-based screening strategies relative to the no-screening strategy per 100,000 men with no prior NPC diagnosis, and all health utility and costs were discount at a 3% annual rate.^c^Their respective ICERs based on the average values of each age.

### Base-case analysis for polygenic risk-stratified screening strategies

**Figure 1:**
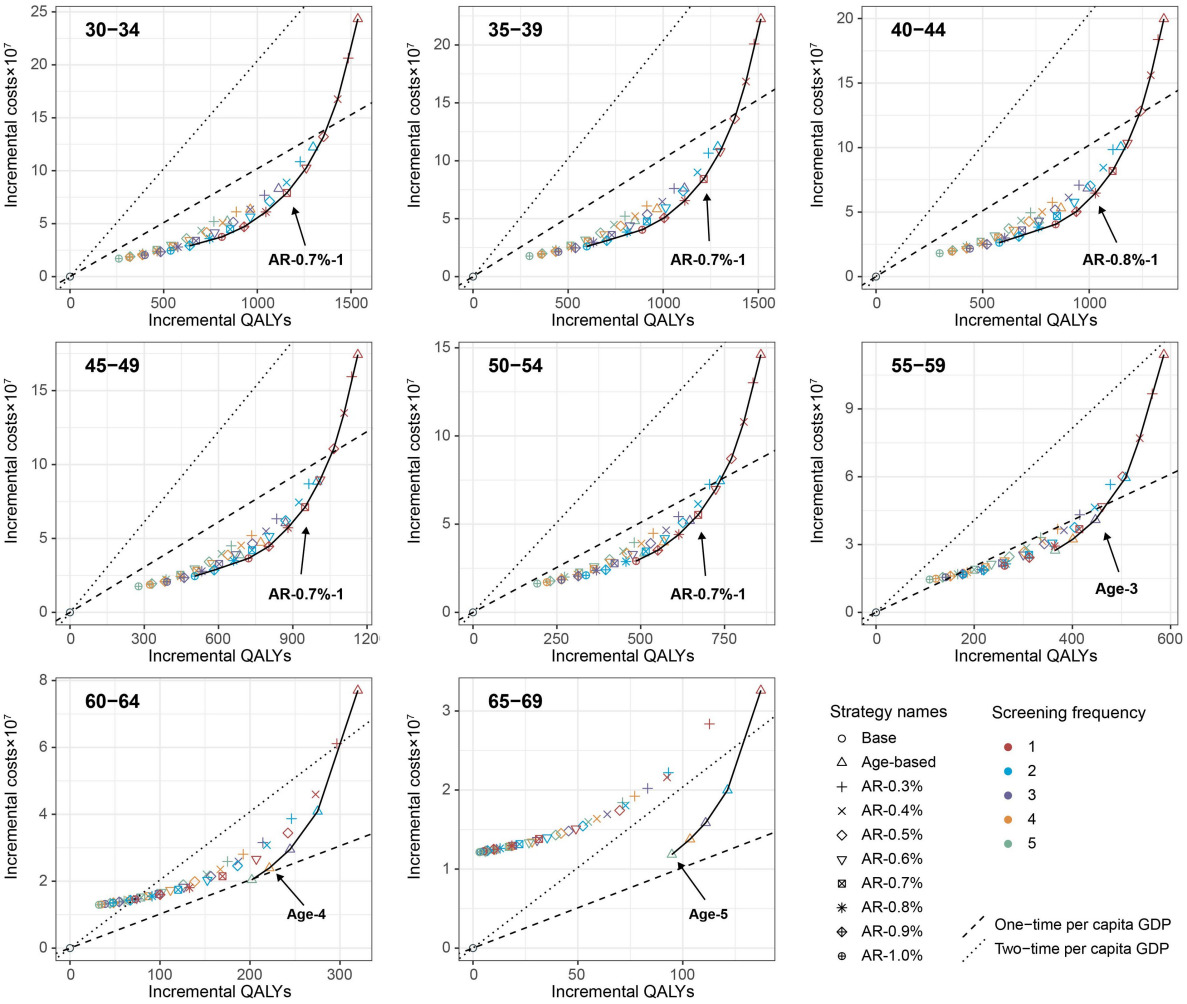
Cost-effectiveness frontier for all screening strategies based on the mean values of each age group under the base-case analysis (100,000 male cohort). Incremental QALYs and incremental costs of screening strategies compared with the no intervention scenario. The line on the each plot was cost-effectiveness efficiency frontier. The strategies on the upper left of the frontier are dominated by the strategies on the lower right of them.

When further considering the polygenic risk-stratified screening strategies into our cost-effectiveness analysis, a total of 46 strategies for men and 31 strategies for women were modeled. For men aged 30–54, the cost-effectiveness efficiency frontiers include 8–12 strategies in different initial screening age groups, with almost all strategies being polygenic risk-stratified screening strategies (Figure 1). Among the strategies on efficiency frontiers, the strategies with a 10-year AR threshold of 0.7% or higher were found to be cost-effective except for the age group of 40–44, where a threshold of 0.8% or higher were cost-effective. Notably, among the cost-effective strategies, the strategy labeled “AR-0.7%-1” (the individuals with a 10-year AR greater than 0.7% were defined as high-risk individuals and then underwent EBV serological test every year) was most cost-effective across 30–54 age groups except for 40–44, with the ICER ranging from ¥159,752 to ¥201,738. And the strategy named “AR-0.8%-1” was the most cost-effective for men aged 40–44, with an ICER of ¥160,315 (Table 2). Among the polygenic risk-stratified strategies, the optimal age-based strategies were strongly dominated for men aged 30–49 and were weakly dominated for men aged 50–54 (Table 2). Compared to optimal age-based strategies, the optimal polygenic risk-stratified strategies can reduce nasopharyngoscopies by 5.1–27.7% while maintaining early-stage detection rate and averted death counts. (Table 3). However, for men aged 55 and above, age-based strategies were more cost-effective than polygenic risk-stratified strategies (Figure 1), and the ICERs and outcomes for these strategies on cost-effectiveness frontiers were shown in Supplementary Tables S10, S11.

**Table 2:** Base-case analysis of age-based and polygenic risk-stratified screening strategies in male population aged 30–54 years.

Starting age	Screening strategy^a^	Incremental costs^b^	Incremental QALYs^b^	ICER^c^	
				vs. no screening	vs. preceding strategy on efficiency frontier
30–34	AR-0.9%-2	29,111,252	639	45,554	45,554
	AR-1.0%-1	37,482,718	811	46,243	48,811
	AR-0.9%-1	47,021,145	929	50,596	80,303
	AR-0.8%-1	61,071,288	1,046	58,404	120,782
	AR-0.7%-1	78,972,887	1,158	68,214	159,752
	Age-3^d^	82,980,500	1,113	74,527	Strongly dominated^e^
	AR-0.6%-1	102,694,374	1,261	81,421	229,064
	AR-0.5%-1	132,088,362	1,353	97,613	319,817
	AR-0.4%-1	167,579,080	1,429	117,286	469,354
	AR-0.3%-1	206,352,582	1,485	138,922	685,294
	Age-1	243,353,757	1,536	158,397	726,027
35–39	AR-1.0%-2	26,017,456	598	43,494	43,494
	AR-0.9%-2	31,056,654	703	44,148	47,869
	AR-1.0%-1	40,427,803	890	45,447	50,356
	AR-0.9%-1	50,657,129	1,005	50,406	88,622
	AR-0.8%-1	65,607,054	1,113	58,921	137,812
	Age-3^d^	76,244,383	1,112	68,583	Strongly dominated^e^
	AR-0.7%-1	84,208,307	1,213	69,434	187,301
	AR-0.6%-1	108,184,259	1,300	83,210	274,483
	AR-0.5%-1	136,316,927	1,375	99,111	373,772
	AR-0.4%-1	168,374,167	1,435	117,333	537,752
	Age-1	222,440,579	1,513	146,990	690,553
40–44	AR-1.0%-2	26,272,897	578	45,450	45,450
	AR-0.9%-2	31,140,790	670	46,465	52,830
	AR-1.0%-1	40,513,150	842	48,097	54,452
	AR-0.9%-1	50,296,222	939	53,547	100,884
	AR-0.8%-1	64,694,829	1,029	62,865	160,315
	Age-3^d^	68,403,058	992	68,923	Strongly dominated^e^
	AR-0.7%-1	81,839,728	1,109	73,772	213,660
	AR-0.6%-1	103,555,194	1,180	87,792	309,351
	AR-0.5%-1	128,187,118	1,241	103,295	401,003
	AR-0.4%-1	156,027,236	1,289	121,069	582,855
	Age-1	199,699,464	1,350	147,916	711,883
45–49	AR-1.0%-2	24,488,537	504	48,582	48,582
	AR-0.9%-2	28,596,500	582	49,104	52,460
	AR-1.0%-1	36,510,587	721	50,624	57,001
	AR-0.9%-1	44,614,497	803	55,537	98,693
	AR-0.8%-1	57,193,406	880	64,966	163,298
	Age-3^d^	60,427,104	871	69,374	Strongly dominated^e^
	AR-0.7%-1	71,157,655	950	74,936	201,738
	AR-0.6%-1	89,760,281	1,011	88,803	303,935
	AR-0.5%-1	110,623,673	1,065	103,836	382,189
	AR-0.4%-1	134,854,703	1,107	121,772	576,057
	Age-1	174,302,204	1,163	149,916	714,166
50–54	AR-1.0%-1	29,092,300	486	59,831	59,831
	AR-0.9%-1	35,177,348	551	63,836	93,889
	AR-0.8%-1	44,147,233	614	71,940	143,250
	Age-3^d^	51,960,292	646	80,381	Weakly dominated^e^
	AR-0.7%-1	55,270,634	671	82,324	192,760
	AR-0.6%-1	69,847,411	724	96,503	278,131
	AR-0.5%-1	87,143,638	771	113,098	370,150
	AR-0.4%-1	107,968,030	808	133,693	561,814
	Age-1	146,080,468	858	170,207	752,188

^a^The screening strategies are labeled as follows: for age-based strategies, “Age-screening frequency”; for polygenic risk-stratified strategies, “AR-10-year NPC absolute risk threshold-screening frequency.” For example, “AR-0.7%-1” indicated that individuals with a 10-year AR exceeding 0.7% undergo an EBV serological test every years.^b^Incremental health benefits gained, and costs incurred from age-based screening strategies relative to the no-screening strategy per 100,000 men with no prior NPC diagnosis, and all health utility and costs were discount at a 3% annual rate.^c^Their respective ICERs based on the average values of each age.^d^The optimal age-based strategy was included for ease of comparison.^e^A strongly dominated strategy was defined as a strategy for which another strategy existed that yielded better health benefit at lower cost, and a weakly dominated strategy was defined as a strategy dominated by a linear combination of 2 other strategies.

**Table 3:** Screening outcomes and utilization rate using polygenic risk-stratified screening strategies in male population aged 30–54 years.

Starting age	Screening strategy^a^	Anti-EBV IgA tests, *n*	Nasal endoscopy tests, *n*	Detected NPC, *n*^b^			NPC deaths averted
				Early stage NPC	Locoregionally advanced NPC	Recurrent NPC	
30–34	Base	—	—	448	662	409	—
	AR-0.9%-2	286,203	11,087	752	441	288	113
	AR-1.0%-1	425,633	16,427	835	375	258	140
	AR-0.9%-1	571,914	22,030	892	330	238	160
	AR-0.8%-1	782,858	30,103	949	285	218	179
	AR-0.7%-1	1,045,769	40,152	1,004	242	200	197
	Age-3^c^	1,204,547	46,314	993	263	201	199
	AR-0.6%-1	1,388,759	53,251	1,055	201	183	215
	AR-0.5%-1	1,805,003	69,132	1,100	165	168	230
	AR-0.4%-1	2,302,560	88,107	1,137	135	156	242
	AR-0.3%-1	2,839,396	108,571	1,165	112	148	251
	Age-1	3,512,767	134,222	1,189	92	140	260
35–39	Base	—	—	422	633	390	—
	AR-1.0%-2	209,379	8,127	675	451	288	94
	AR-0.9%-2	279,877	10,844	722	415	271	110
	AR-1.0%-1	416,232	16,066	802	352	242	137
	AR-0.9%-1	555,361	21,396	856	310	223	155
	AR-0.8%-1	755,061	29,039	908	269	205	172
	Age-3^c^	1,051,690	40,481	941	255	193	189
	AR-0.7%-1	1,000,095	38,406	957	230	188	189
	AR-0.6%-1	1,314,309	50,408	1,002	194	173	204
	AR-0.5%-1	1,682,774	64,469	1,043	162	161	217
	AR-0.4%-1	2,108,130	80,695	1,076	135	150	228
	Age-1	3,038,922	116,174	1,122	99	137	244
40–44	Base	—	—	381	576	354	—
	AR-1.0%-2	191,076	7,420	613	410	260	84
	AR-0.9%-2	252,829	9,800	654	379	245	99
	AR-1.0%-1	375,373	14,493	726	322	219	122
	AR-0.9%-1	496,701	19,142	773	285	203	138
	AR-0.8%-1	671,939	25,851	818	250	187	153
	Age-3^c^	886,031	34,130	843	242	178	166
	AR-0.7%-1	880,840	33,840	862	216	173	168
	AR-0.6%-1	1,146,199	43,979	902	185	159	181
	AR-0.5%-1	1,451,345	55,626	939	156	148	193
	AR-0.4%-1	1,803,445	69,060	968	134	140	203
	Age-1	2,572,261	98,371	1,009	101	127	217
45–49	Base	—	—	334	518	317	—
	AR-1.0%-2	153,591	5,974	532	377	236	71
	AR-0.9%-2	202,446	7,859	568	352	223	84
	AR-1.0%-1	297,260	11,493	627	307	202	103
	AR-0.9%-1	391,524	15,108	667	275	188	116
	AR-0.8%-1	533,006	20,529	707	245	174	130
	Age-3^c^	736,845	28,411	743	227	161	147
	AR-0.7%-1	694,293	26,701	746	214	162	143
	AR-0.6%-1	907,290	34,844	783	186	149	156
	AR-0.5%-1	1,151,763	44,179	816	160	139	167
	AR-0.4%-1	1,441,012	55,220	843	139	130	176
	Age-1	2,109,951	80,726	883	110	119	190
50–54	Base	—	—	274	429	260	—
	AR-1.0%-1	193,337	7,488	483	279	178	73
	AR-0.9%-1	259,763	10,039	517	254	166	84
	AR-0.8%-1	355,383	13,708	552	228	154	96
	Age-3^c^	589,892	22,749	605	197	136	118
	AR-0.7%-1	475,779	18,319	586	202	142	108
	AR-0.6%-1	633,057	24,336	620	177	131	119
	AR-0.5%-1	823,002	31,593	650	154	121	129
	AR-0.4%-1	1,055 517	40,471	676	135	114	138
	Age-1	1,652,872	63,250	713	106	103	151

^a^The screening strategies are labeled as follows: for age-based strategies, Age-screening frequency; for polygenic risk-stratified strategies, AR-10-year NPC absolute risk threshold-screening frequency.^b^Early stage NPC includes those in stage I and stage II; Locoregionally advanced stage NPC includes those in stage III and stage IVA-B; Recurrent NPC includes those who were *de novo* metastasis and those who developed local relapse, regional relapse or metastasis after treatment.^c^The optimal age-based strategy was included for ease of comparison.

Similarly, for women aged 30–54, the polygenic risk-stratified strategies labeled “AR-0.30%-1” were found to be the most cost-effective, with ICERs ranging from ¥145,069 to ¥178,223 (Supplementary Figure S11; Supplementary Table S12). The optimal age-based strategies were strongly dominated in three out of five age groups (30–34, 40–44, and 45–49), and were weakly dominated in the remaining two age groups (35–39 and 50–54, Supplementary Table S12). The optimal polygenic risk-stratified strategies can reduce the number of EBV serological tests and nasopharyngoscopy required by 39–54% for the women aged 30–54, while still achieving comparable numbers of early diagnosis and averted death compared to the optimal age-based screening strategies (Supplementary Table S13). For women aged 55 and above, polygenic risk-stratified strategies were also not cost-effective (Supplementary Table S12).

### Sensitivity analysis

**Figure 2:**
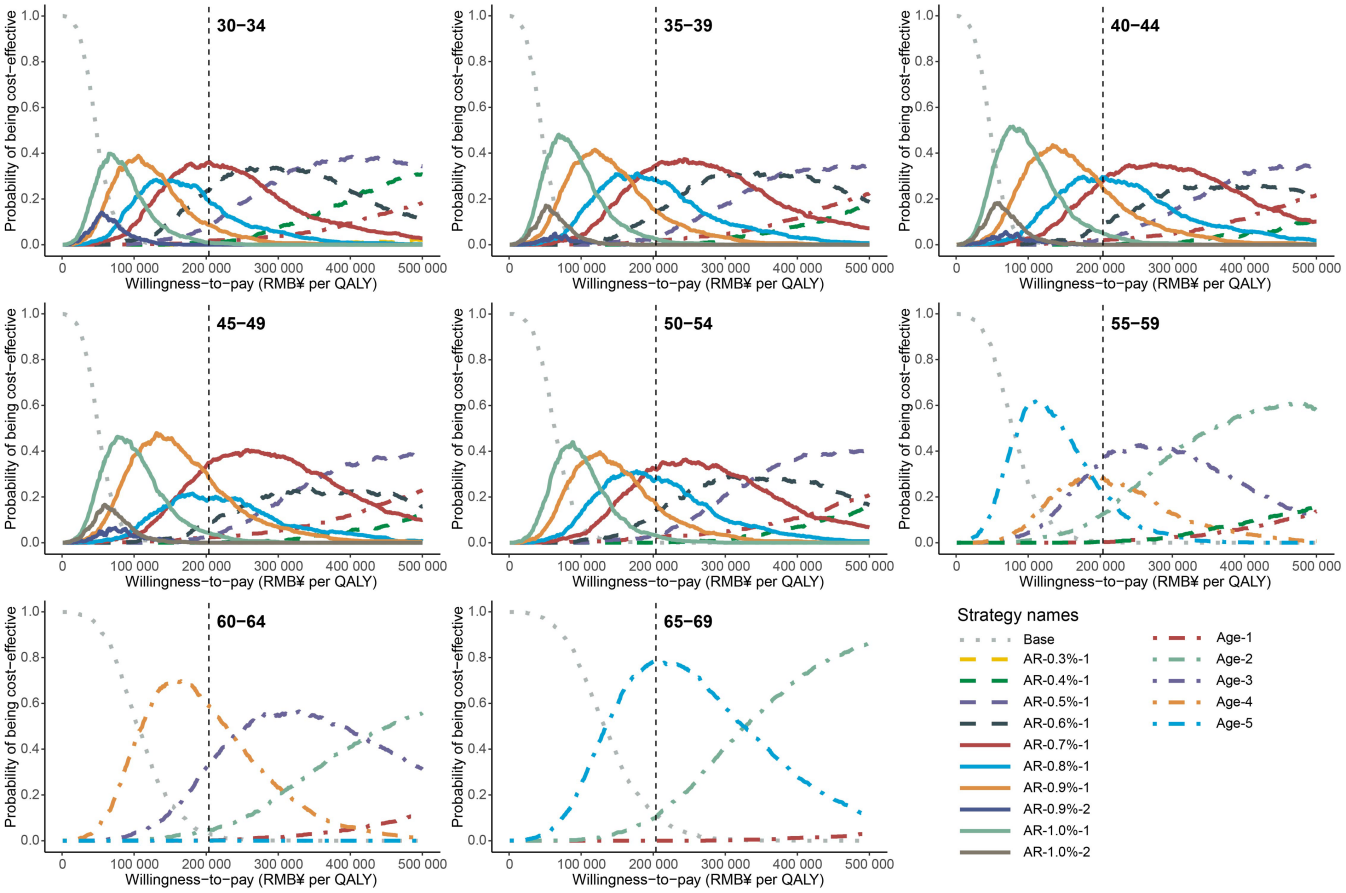
Cost-effectiveness acceptability curves for all strategies on the cost-effectiveness efficiency frontier in male population. The gray dotted line represents the curves of no screening scenario, the dot dash lines represent the curves of age-based strategies, and the solid lines represent the curves of polygenic risk-stratified strategies. The vertical gray dash line represents the WTP of twice *per capita* GDP. The screening strategies are labeled as follows: for age-based strategies, Age-screening frequency; for polygenic risk-stratified strategies, AR-10-year NPC absolute risk threshold-screening frequency.

In order to evaluate the robustness of our findings, we conducted probabilistic sensitivity analyses by simultaneously varying crucial input parameters. The cost-effectiveness acceptability curve of male population was shown in Figure 2, which depicts the probability of each strategy on the cost-effectiveness efficiency frontiers being considered cost-effective at different WTP thresholds, ranging from ¥0 to ¥500,000. For men aged 30–54, the optimal polygenic risk-stratified strategies showed a top probability (29.4–35.8%) of being cost-effective at a WTP of twice the *per capita* GDP. For men aged 55–59, 60–64, and 65–69, the optimal age-based strategies had top probabilities of 36.5, 58.9, and 78.1%, respectively. Similar results were observed for women. Specifically, for women aged 30–54, the optimal polygenic-risk stratified strategy labeled “AR-0.30%-1” showed top probabilities ranging from 33.5 to 46.8%. For women aged 55 and above, the no screening strategy displayed the highest probabilities, ranging from 69.4 to 98.4% (Supplementary Figure S12). The median and 95% confidence interval of the ICER resulting from probabilistic sensitivity analyses were consistent with the results of base-case analyses, further demonstrating the robustness of the optimal strategies (Supplementary Table S14).

**Figure 3:**
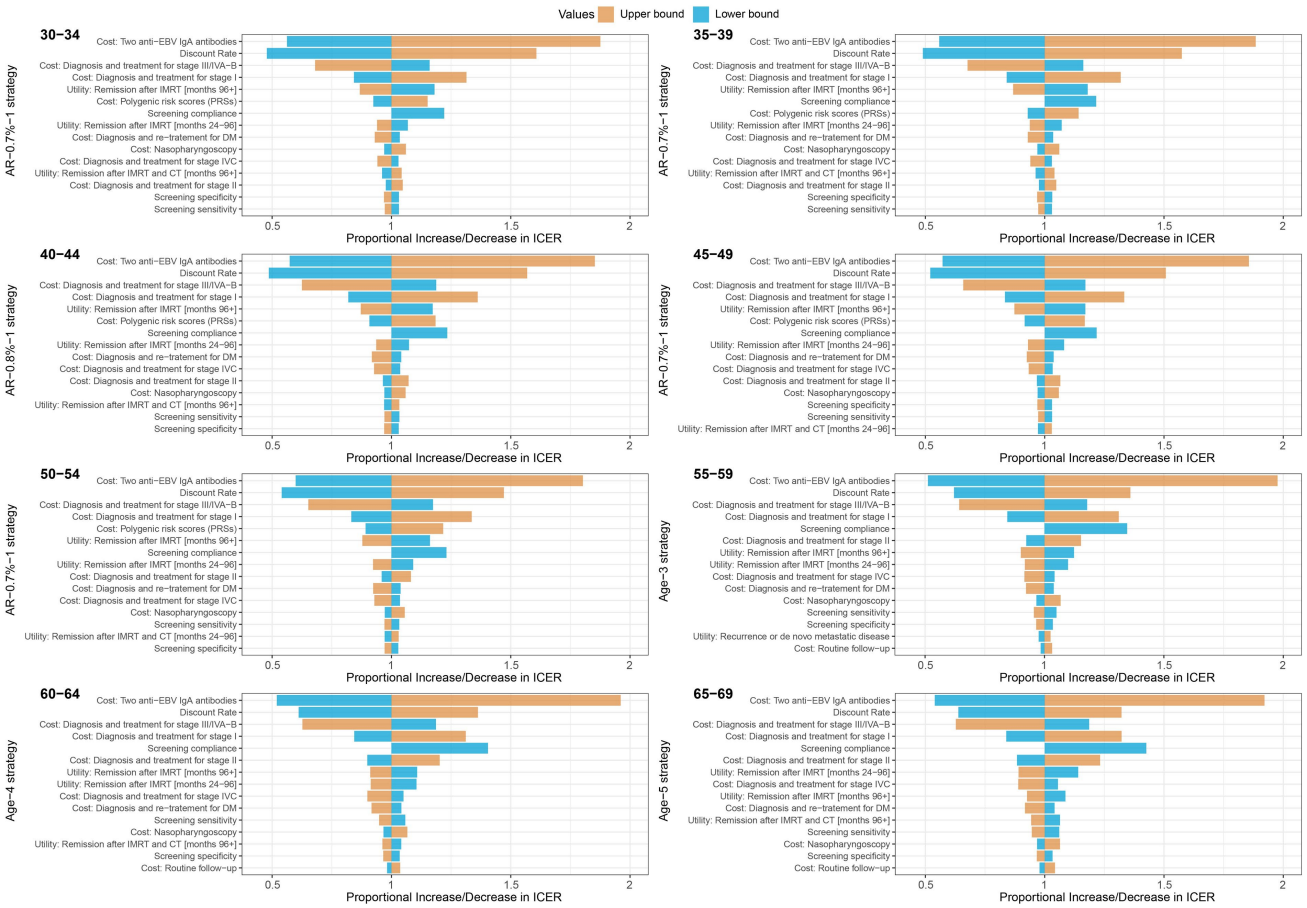
Tornado plots for one-way deterministic sensitivity analysis of the optimal strategies compared with the no screening strategy in male population. Compared to no screening strategy, a proportional increase or decrease in the incremental cost-effectiveness ratio is plotted for upper (orange) and lower (blue) bounds of model parameters. The optimal strategies were polygenic risk-stratified strategies for 30–34, 35–39, 40–44, 45–49 and 50–54 age groups, and the age-based strategies were optimal for 55–59, 60–64 and 65–69 age groups.

Furthermore, we conducted univariate sensitivity analyses on the optimal screening strategies for different age groups and genders. We varied the input parameters within upper bound and lower bound as shown in Supplementary Table S1 and assessed the proportional increase or decrease in ICER compared to the base-case values. Ultimately, we identified and presented the top 15 sensitive parameters. According to our results, the costs of testing two anti-EBV IgA antibodies and discount rate were top two parameters exhibiting the highest sensitivity to the ICER in the optimal screening strategies for both men and women (Figure 3; Supplementary Figure S13). These parameters, including the costs of diagnosis and treatment for stage I and stage III/IVA-B NPC, the health utility associated with stage I NPC, and compliance, were also important parameters that impact the results of ICER. In addition, the cost of PRS was found to be sensitive to the ICER in the optimal strategies for individuals aged 30–54, with values ranging from 0.89 to 1.22 for men and from 0.79 to 1.42 for women.

## Discussion

Our study highlights the potential benefits of incorporating polygenic data into population-level screening for nasopharyngeal carcinoma in endemic areas of China. The polygenic risk-stratified screening strategies proved to be more cost-effective than age-based screening strategies, particularly for the population aged 30–54 years. The polygenic risk-stratified screening strategies not only reduce the numbers of EBV serological tests and nasopharyngoscopies but also maintained most of the early diagnoses and averted deaths achieved by age-based screening strategies. Almost all strategies on the cost-effective efficiency frontiers were polygenic risk-stratified screening strategies, indicating their superior performance in terms of cost-effectiveness. For men, the polygenic risk-stratified screening strategy with a 0.7% or 0.8% 10-year AR threshold and a 1-year screening frequency was found to be the most cost-effective. Similarly, for women, the strategy with a 0.3% risk threshold and a 1-year screening frequency was the most cost-effective. These strategies yielded more QALYs at a lower cost level, making them attractive choices for targeted screening. Overall, our findings may offer valuable insights for personalized NPC screening, potentially leading to better outcomes and resource allocation in endemic areas of China.

Recently, polygenic risk scores have shown promise in predicting an individual’s genetic susceptibility to cancer and have the potential to improve the efficiency of population-level screening (13, 37–39). However, the economic feasibility of incorporating polygenic data into population-level screening is a critical consideration (13, 40, 41). Several studies have focused on cancers like breast, prostate, and colorectal cancer, using various model to evaluate the use of PRS in cost-effectiveness analyses of cancer screening. These studies have suggested that using polygenic risk data would be cost-effective (14, 16, 17, 42–46). Our study aligns with these findings and suggests that polygenic risk-stratified screening strategies could be more cost-effective for NPC, particularly for the population aged 30–54 years. This is because, firstly, polygenic risk stratification allows for a more precise estimation of an individual’s risk for NPC, thus enabling tailored screening strategies. Secondly, targeting screening efforts toward individuals with a high genetic predisposition to NPC allows for more efficient allocation of healthcare resources. This maximizes the benefits of screening while minimizing unnecessary interventions and associated costs for individuals at low genetic risk. Finally, since the SNPs composing PRS only require one-time testing in a lifetime, the cost of assessing genetic risk for all participants is lower than the cumulative cost of EBV antibody testing for low-risk individuals.

Implementing this risk-stratified screening strategy involves prioritizing more frequent screenings for individuals identified as high risk, with the goal of maximizing the identification of NPC patients within this high-risk population. While individual PRS was innate and constant, the 10-year AR value we calculate by combining age and PRS are dynamic and effectively pinpoint individuals at high risk across age groups. By setting a higher predefined 10-year AR threshold, the higher PPV of EBV serological screening for fewer high-risk individuals leads to a higher yield of QALYs per EBV serological test. However, it failed to identify potential early-stage NPC patients in the low-risk population, leading to their progression to the advanced stage before diagnosis, which incurs additional economic costs. To address it, we have developed a set of risk threshold gradients to determine the ideal balance between benefits and costs of screening. In more detail, for men aged 30–54 years, the strategies with a 10-year risk threshold of 0.7% or greater were found to be cost-effective. The strategy with a 0.7% risk threshold and a screening frequency of 1 year (AR-0.7%-1) was most cost-effective, except for the age group of 40–44 where a 0.8% AR threshold (AR-0.8%-1) was most cost-effective. For women aged 30–54 years, the strategy with a 0.3% risk threshold and a 1-year screening frequency (AR-0.3%-1) was the most cost-effective among the considered strategies on the efficiency frontiers. However, for individuals aged 55 and above, the age-based screening strategies were found to be more cost-effective than the polygenic risk-stratified screening strategies. This implies that the polygenic risk-stratified screening strategy may be more beneficial for younger individuals compared to the age-based strategy. Initiating age-based screening for the younger population often results in many unnecessary screening tests and clinical examinations owing to their low incidence rate. But the polygenic risk-stratified screening strategy offers a solution by targeting high-risk individuals to undergo screenings. This approach can effectively offset the additional cost of evaluating the genetic risks of all participants.

In addition to evaluating the cost-effectiveness of polygenic-risk stratified screening strategies, we have also supplemented the existing screening strategies for population with difference age group. In previous health economic evaluations of NPC screening strategies, the focus has primarily been on one-time lifetime screening for individuals aged 50 years (30, 31, 47). However, there is limited understanding of both the optimal screening frequency and the potential benefits of screening individuals in different age groups (6). In this study, we conducted a comprehensive evaluation of the age-based screening strategies in different age groups and explored the optimal screening frequency for each age group. Under a WTP threshold of twice the *per capita* GDP, the optimal screening frequency for men aged 30–59 was found to be every 3 years, while for individuals aged 60 and above, the screening frequency increased to every 4–5 years. It was observed that for younger age groups, more frequent screening led to the detection of a greater number of young patients, yielding higher benefits. For women aged 30–54, the optimal screening frequency was determined to be every 5 years, considering their lower incidence rate. For women aged 55 and above, it was not considered cost-effective to use aged-based screening strategies with a frequency of every 1–5 years. It should be noted that these results were based on a screening strategy set at every 1–5 years. Further study should consider exploring lower frequency screenings.

In sensitivity analysis, the probability sensitivity analysis consistently showed that the optimal screening strategies for each age group had the highest probability of being cost-effective with the WTP threshold, and supported the effectiveness of the proposed screening strategies. Univariate sensitivity analysis identified several model parameters that were sensitive to the results, such as the discount rate, compliance, utility of stage I, and cost of testing anti-EBV IgA antibodies, which were also reported as sensitivity parameters in previous studies (30, 31). Furthermore, we have also assessed the influence of the cost of PRS on ICER in the polygenic risk-stratified strategies, taking into account the lack of accuracy data on per-individual costs of polygenic risk stratification for implementation in large-scale screening programs before. We found the cost of PRS was a crucial sensitive parameter, and its change had a more pronounced impact on women because the cost of PRS for all participants accounts for a larger proportion of the total cost of screening. Additionally, endoscopic compliance is also a sensitive parameter, emphasizing the importance of raising health awareness and promoting screening compliance among the population.

According to the latest expert consensus, the current recommendations for NPC screening involve either male-only or sex-neutral middle-aged adults (6). Our study findings supported that when implementing the same screening strategy, the ICER was generally lower for men due to their higher incidence rate. This indicated better cost-effectiveness for men, which aligns with the previous study (30, 31). However, if screening was conducted for the general population without considering gender and utilizing a unified screening strategy, there were certain challenges in terms of cost-effectiveness. Therefore, our study constructed multi screening Markov models with different risk threshold criteria and screening frequencies based on the disease risk among different genders and ages. Our results showed that the risk threshold for women was lower than that for men in the optimal polygenic risk-stratified strategies, and the screening frequency for women was also lower than that for men in the optimal age-based strategies. It was essential to utilize different screening criteria tailored to specific gender populations. These proposed screening strategies in our study offered more flexible options for NPC screening in areas with resource disparities, with the aim of achieving more effective personalized screening.

Our study also has several limitations that should be acknowledged. Firstly, we acknowledged that our research model is tailored for the high-risk endemic areas, especially for the Southern China region. This aligned with the expert consensus recommending mass screening exclusively in endemic areas (6). Therefore, our Markov models and conclusions may not be directly applicable to non-endemic area with different cancer incidence rate, genetic backgrounds and healthcare system. Secondly, in comparison to existing screening guidelines that focus solely on age in endemic areas (5), we have integrated sex and genetic factors to refine the precision of screening. However, other factors associated with NPC risk, such as smoking, should be taken into account in further research. Then, our age-based screening strategy did not consider the past EBV serological results, as the unpredictable nature of anti-EBV IgA changes in lifetime, making it challenging to accurately simulate the existing screening protocol (48, 49). Moreover, incorporating compliance into modeling is indeed complex, as it can vary based on age, sex, education, socioeconomic status and other factors (8). We assumed a 100% compliance rate for further nasopharyngeal endoscopy, which may have led to an overestimation of the health benefits and costs associated with NPC screening. We conducted univariate sensitivity analyses using assumptions of 70% for compliance (7). Finally, our model did not consider the impact of contemporary treatment modalities, such as the increasingly widespread use of immunotherapy (50–52).

## Conclusion

In conclusion, our study provides compelling evidence supporting the superiority of polygenic risk-based NPC screening strategies over age-based strategies for individuals aged 30–54, from a health economics perspective. By tailoring the screening frequency and risk stratification based on age, sex and polygenic risk score, we can optimize the use of resources and maximize the health benefits for NPC screening.

## Data availability statement

The original contributions presented in the study are included in the article/Supplementary material, further inquiries can be directed to the corresponding authors.

## Ethics statement

The study was approved by the ethics committee of the Sun Yat-sen University Cancer Center. The patients/participants provided their written informed consent to participate in this study.

## Author contributions

D-WY: Conceptualization, Data curation, Investigation, Methodology, Visualization, Writing – original draft, Writing – review & editing, Formal analysis, Software. JM: Methodology, Writing – review & editing, Supervision. W-QX: Writing – review & editing, Data curation, Resources. MT: Data curation, Writing – review & editing. LL: Data curation, Writing – review & editing. YZ: Writing – review & editing, Data curation. HD: Resources, Writing – review & editing. T-MW: Resources, Writing – review & editing. YL: Resources, Writing – review & editing. Y-XW: Resources, Writing – review & editing. X-HZ: Resources, Writing – review & editing. TZ: Resources, Writing – review & editing. X-ZL: Resources, Writing – review & editing. P-FZ: Resources, Writing – review & editing. X-YC: Resources, Writing – review & editing. XY: Resources, Writing – review & editing. FL: Resources, Writing – review & editing. MJ: Resources, Writing – review & editing. YS: Resources, Writing – review & editing. Y-QH: Resources, Conceptualization, Data curation, Formal analysis, Methodology, Supervision, Writing – original draft, Writing – review & editing. W-HJ: Conceptualization, Data curation, Methodology, Resources, Supervision, Writing – original draft, Writing – review & editing, Funding acquisition, Investigation, Visualization.
