# Supplementary material for: Polygenic risk-stratified screening for nasopharyngeal carcinoma in high-risk endemic areas of China: a cost-effectiveness study
Source: Front Public Health. 2024 May 2;12:1375533. doi: 10.3389/fpubh.2024.1375533 (PMC11097958; doi:10.3389/fpubh.2024.1375533)
Supplement: Supplementary file 1 [file Data_Sheet_1.docx]

**Supplementary material - Polygenic risk-stratified screening for nasopharyngeal carcinoma in high-risk endemic areas of China: A cost-effectiveness study**

**Supplementary Methods.**

**Supplementary Tables**

**Supplementary Table 1.** Parameters used in the Markov model.

**Supplementary Table 2.** Performance characteristics for polygenic risk-stratified NPC screening strategies in individuals aged 50.

**Supplementary Table 3.** Age-specific background mortality rate.

**Supplementary Table 4.** Stage- and age-specific incidences rates of NPC for Asian and Pacific Islander from the SEER-18 dataset and its proportions of each stage in different age groups.

**Supplementary Table 5.** The characteristics of non-metastatic NPC in Sun Yat-sen University Cancer Center.

**Supplementary Table 6.** The parameters of estimating recurrence and survival for NPC.

**Supplementary Table 7.** Model validation of the Markov model-predicted survival compared with the real-world observed survival for non-metastatic NPC.

**Supplementary Table 8.** The costs of items in screening, diagnosis and treatment of NPC.

**Supplementary Table 9.** Base-case analysis of only age-based screening strategies in female population.

**Supplementary Table 10.** Base-case analysis of age-based and polygenic risk-stratified screening strategies in male population aged 55-69 years.

**Supplementary Table 11.** Screening utilization and outcomes using the screening strategies on cost-effectiveness efficiency frontiers in male population aged 55-69 years.

**Supplementary Table 12.** Base-case analysis of age-based and polygenic risk-stratified screening strategies in female population.

**Supplementary Table 13.** Screening utilization and outcomes using the screening strategies on cost-effectiveness efficiency frontiers in female population.

**Supplementary Table 14**. The results of optimal screening strategies in probabilistic sensitivity analysis

**Supplementary Figures**

**Supplementary Fig 1.** Markov model of NPC natural history in high-risk endemic areas.

**Supplementary Fig 2.** Flowchart of screening by three strategies.

**Supplementary Fig 3.** The cumulative distribution of the 10-year absolute risk.

**Supplementary Fig 4.** The proportion of high risk population in general population (blue) or NPC patients (orange) of each age group from 30 to 69 years old.

**Supplementary Fig 5.** The transition probability distributions in the transition probability matrix between undetected states and detected states of our Markov model.

**Supplementary Fig 6.** Stage- and age-specific observed incidence rate of NPC for Guangzhou registry data of CI5 (orange) and estimated incidence rate of NPC from Markov model (blue).

**Supplementary Fig 7.** The actual and simulated stage-specific local relapse-free survival (LRFS), regional relapse-free survival (RRFS) and distant metastasis-free survival (DMFS) for non-metastatic NPC.

**Supplementary Fig 8.** The actual and simulated stage-specific overall survival.

**Supplementary Fig 9.** Cost-effectiveness frontiers for only age-based strategies based on the mean values of each age under the base-case analysis (100 000 male cohort members).

**Supplementary Fig 10.** Cost-effectiveness frontiers for only age-based strategies based on the mean values of each age under the base-case analysis (100 000 female cohort members).

**Supplementary Fig 11.** Cost-effectiveness frontiers for all screening strategies based on the mean values of each age under the base-case analysis (100 000 female cohort members).

**Supplementary Fig 12.** Cost-effectiveness acceptability curves for all strategies on the cost-effectiveness efficiency frontier in female population.

**Supplementary Fig 13.** Tornado plots for one-way deterministic sensitivity analysis of the optimal aged-based strategies and the optimal polygenic risk-stratified strategies compared with the no screening strategy in female population.

**Supplementary Methods**

**Markov Decision-analytic Model**

The model was similar to the Markov model described by Miller et al.^1^, and included 15 health states included perfect health, five separate stages of undetected NPC (preclinical stage I, II, III, IVA/B, IVC), five separate stages of detected NPC (clinical stage I, II, III, IVA/B, IVC), three separate prognostic states (local recurrence, regional recurrence, and distant metastasis), and death (**Supplementary Fig 1**). The individuals who developed NPC entered a state of undetected stage I, which could progress to more advanced stages of undetected disease. And each undiagnosed stage of NPC could manifest symptomatically and was diagnosed in clinic, as observed in unscreened populations with stage-specific incidence rates. At the time of onetime screening, a true positive (based on the sensitivity of two anti-EBV IgA antibodies screening strategy) resulted in immediate detection at that stage. However, false negatives and unscreened cases would continue to progress until usual symptomatic detection. If a positive result was obtained (whether it was a true positive or false positive), the individual would be examined nasopharyngeal endoscopy. Each non-metastatic stage of NPC (I-IVB) underwent definitive therapy and entered remission with no evidence of disease, and then could either die from background mortality or develop a recurrence (included local relapse, regional relapse and distant metastasis) prior to death from NPC. Additionally, there was also a background mortality rate and the opportunity to remain in the same state for each given state.

**Polygenic Risk Profiles**

To construct a polygenic risk-stratified screening Markov model, it is essential to first determine the proportion of high-risk individuals above a specific threshold in the general population and the incidence rate of nasopharyngeal carcinoma (NPC) among this high-risk population. To estimate the proportion of high-risk individuals in the general population, different 10-year absolute risk (AR) thresholds can be defined to determine the proportion of high-risk individuals in different age groups of the natural population in high-risk endemic areas of NPC. For instance, assuming a 10-year AR threshold of 0.5% for high-risk males, the cumulative frequency distribution curve of the 10-year AR values in 50-year-old males from the general population can be used to determine that high-risk individuals account for 71.38% of the general population (**Supplementary Fig 3A**). The incidence rate of NPC among high-risk individuals can be understood as the proportion of high-risk individuals among NPC patients because the incidence rate of NPC in each age group of the general population is known, and the number of high-risk individuals with NPC is derived from the number of high-risk individuals who develop NPC. By exploring the correlation between PRS and 10-year AR in general population, we could determine the 10-year AR for 4506 NPC patients by utilizing the PRS calculated from our previous study. Based on the cumulative frequency distribution curve of 10-year AR values in NPC patients (**Supplementary Fig 3B**), we can estimate that high-risk individuals account for 86.46% of NPC patients. As described above, the proportions of high-risk individuals in general population and in NPC patients of each age from 30 to 69 years old were calculated respectively (**Supplementary Fig 4**). And the 10-year AR thresholds were defined based on the proportion of high-risk individuals in the general population at the age of 50, which is associated with the highest 10-year AR value. Following discussions with experts, a proportion ranging from 20% to 90% was deemed reasonable. As a result, the final 10-year AR threshold is set at 0.3% to 1.0% for males and 0.1% to 0.3% for females. The detailed performance characteristics for the polygenic risk-stratified NPC screening strategies with different 10-year AR thresholds in individuals aged 50 were shown in **Supplementary Table 2**.

**Transition Probabilities**

Background mortality was derived from the WHO global health observatory age- and sex-specific death rate in 2019 (**Supplementary Table 3**)^2^. The incidence rates of NPC were based on Guangzhou registry data of CI5, which represents areas with high incidence of NPC in China^3^.

As previous described, we related the undiagnosed prevalence of NPC with observed incidence rates^1^. The case-weighted ratio R of prevalent-to-incident NPC cases was calculated to be 4.0, the undiagnosed stage-, age-, and sex-specific prevalence of NPC was the observed age- and sex-specific incidence rate from Guangzhou registry data multiplied by the pooled prevalence-to-incidence ratio. And the stage distribution of undiagnosed NPC were assumed to the distribution of screen-detected cases, which stage I, II, III, IVA/B, and IVC NPC was 34.9%, 38.1%, 23.0%, 4.0% and 0.0% separately.

The transition probability of perfect health to undetected stage I NPC was weighted by an age-specific coefficient α, which considered age-specific incidence rates and death rates, and varied over time.

$$\alpha=\frac{R}{5}\left[ \left( 1-{Mortality}_{i} \right)^{5}\times\frac{{Incidence}_{i+1}}{{Incidence}_{i}}-1 \right]+1$$

*R*: the ratio of prevalent-to-incident NPC cases.

*Mortality_i_*: the mortality rate of *i*th age group.

*Incidence_i_*: the incidence rate of *i*th age group

*Incidence_i+_*_1_: the incidence rate of *i*+1th age group

To estimate the transition probabilities among undetected stages of NPC and the stage-specific probability of symptomatic presentation (undetected to detected NPC), we initiated the analysis by generating 1000 sets of random transition probabilities bounded from 0-1 and modeled the natural history of NPC from development until symptomatic presentation. Subsequently, we calibrated the transition probabilities using three different optimizers (bobyqa, nlminb and Rvmmin) through the R package "optimax"^4^. Furthermore, based on prior knowledge of the transition among the states of NPC in short times, we known that: 1) the transition probability between adjacent stages is greater than between non-adjacent stages; 2) the transition probability decreases as the number of stages crossed increases; and 3) the higher the stage, the more obvious the symptoms, and the higher the transition probability from undetected to detected NPC. We filtered out sets that do not comply with prior knowledge and obtained the transition probability distribution between each state, as shown in **Supplementary Fig 5**. We then selected the median of the transition probability distribution as the transition probability for the Markov base case analysis, while used the interquartile range to establish a range for each state transition probability. To validate the transition probabilities, we calculated the stage-specific incidence rate in each age group of the Markov base case, compared it with the observed incidence rate, and confirmed their accuracy. As there was a lack of stage-specific incidence rates for each age group of NPC in the Chinese population, we computed the proportions of each stage in different age groups by utilizing the stage-specific incidence rates from the SEER database in Asian and Pacific Islander populations (**Supplementary Table 4**)^1^. We finally estimated the stage-specific incidence rates in different age groups by referring to the incidence rate from the Guangzhou site of the five-continent cancer registry^3^. These estimated stage-specific incidence rates were considered as the observed incidence rates of NPC. The **Supplementary Fig 6** showed that the incidence rates of NPC simulated by the Markov model were in close agreement with the observed incidence rates, thereby confirming the accuracy of the model's transition probability evaluation.

**Recurrence and Survival Estimates**

The recurrence and survival for NPC were estimated using the NPC-specific database from the big-data platform of Sun Yat-sen University Cancer Center (SYSUCC), which includes 10 126 newly diagnosed non-metastatic NPC patients admitted between 2009 and 2015^5^. All patients received IMRT. During a median follow-up period of 67 months, 707 cases of local recurrence, 559 cases of regional recurrence, 1333 cases of distant metastasis, and 1603 deaths were observed. The characteristics of NPC patients are shown in the **Supplementary Table 5**.

The transition probabilities of detected NPC to LR, RR, and DM were fitted by parametric survival models using the distributions of exponential, gompertz, log-logistic, log-normal and Weibull^6^. Specifically, the log-normal distribution was applied to the LR and RR data, while the gompertz distribution was applied to the DM data, as they demonstrated the best fit based on the Akaike information criterion^6,7^. The transition probabilities of detected NPC to death (non-recurrences-related mortality), and LR, RR, DM to death, were estimated using the same method as described above (**Supplementary Table 6**). For detected NPC, more than 95% recurrences and death occur within 96 months based on our database, so the transitions of detected NPC to recurrences and death were considered to have occurred within 96 months. And owing to the occurrence of more than 95% of death within 60 months for the patients of LR, RR, and DM, we also consider the transition to occur within 60 months. We used a Markov model to simulate the natural history of prognosis for 45-year-old NPC patients across different stages, in order to validate our predicted probabilities of metastasis. The age was same with the median age of patients in database, and the male-to-female ratio of the simulated patients in was 2:1, which was also consistent with the ratio in our database. And we compared the simulated overall survival rates for each stage with the real-world overall survival rates (**Supplementary Table 7**), and the survival curves were presented in **Supplementary Fig 7**.

To estimate the survival rate of patients with de novo metastatic NPC, we acquired the survival curve from a previous clinical study that included 216 NPC patients who underwent palliative chemotherapy at Sun Yat-sen University Cancer Center from 2008 to 2015^8^. And we used the software "Get Data Graph Digitizer" (http://getdata-graph-digitizer.com/) to extract the survival rates from the overall survival (OS) curve of the palliative chemotherapy group. Then, we reconstructed individual data using the "survHE" package and built the original survival curve^9^. Finally, we utilized parameter distribution to fit the survival curve, and the log-normal distribution produced the best fit according to the AIC criterion (**Supplementary Fig 8**).

**Cost**

The total costs of anti-VCA/EBNA IgA in China has been estimated in previous study, which includes costs of sample collection, transportation, laboratory resources and personnel^1^. We have previously reported the costs of reagents and consumables for the PRS test, and in this study, we also accounted for the transportation and personnel costs in the total costs of the PRS^1,10^. The cost of nasopharyngeal endoscopy was derived from Medical Insurance Administration Bureau of Guangzhou, China^11^.

The diagnostic workup for NPC were based on the Guidelines of Chinese society of clinical oncology (CSCO) and includes routine clinic visits, plasma cfEBV DNA load testing, needle biopsy, pathology review, and imaging tests^12^. Imaging modalities included MRI scans of the head and neck, CT scans of the chest and abdomen, and bone scintigraphy. In particular, NPC patients undergo at least two MRI scans of the head and neck (one upon admission and another at discharge). High-risk NPC patients (stage III-IV) undergo PET/CT imaging as part of their diagnostic evaluation. Clinical staging was based on the American Joint Committee on Cancer (AJCC) 7^th^ edition staging system. The patients with stage I received intensity-modulated radiotherapy (IMRT) only, while patients with stage II received concurrent chemoradiotherapy (CCRT). Patients with stage III-IVA/B underwent induction chemotherapy followed CCRT and those with stage IVC underwent indefinite palliative chemotherapy. For all non-metastatic NPC patients, a total dose of 66-70 Gy was administered, with a clinical target dose of 54-60 Gy delivered in 30-35 fractions. Cisplatin was administered at a dose of 100 mg/m^2^ on days 1, 22, and 43 during CCRT. The GP regimen, consisting of gemcitabine at a dose of 1 g/m^2^ on day 1 and 8 and cisplatin at a dose of 25 mg/m^2^ on days 1-3, every three weeks for three cycles, was used for induction chemotherapy. For newly diagnosed metastatic NPC patients, indefinite palliative chemotherapy with the GP regimen was administered at a dose of gemcitabine 1 g/m^2^ on day 1 and 8 and cisplatin 80 mg/m^2^ on days 1-3, every three weeks for six cycles (**Supplementary Table 8**).

After completing treatment, patients underwent a minimally intensive surveillance program that was administered in accordance with RTOG guidelines (every 3 months in year 1; every 6 months in year 2; every 12 months in years 3-5, 10 visits in total)^13,14^. In the event of disease recurrence, treatment options included endoscopic nasopharyngectomy or re-irradiation for local recurrence, neck dissection or re-irradiation for neck recurrence, and chemotherapy or chemoradiotherapy for distant metastasis^15^. We assumed a discount rate of 3% (range 0-5) for both quality-adjusted life-years (QALYs) and costs.

**Supplementary Table 1. Parameters used in the Markov model**.

| **Parameter** | **Base-case estimate** | **Lower Bound** | **Upper Bound** | **Distribution** | **Reference** |
| --- | --- | --- | --- | --- | --- |
| Transition Probability |  |  |  |  |  |
| Each undetected NPC stage to more advanced stages, as well as stage-specific probability of undetected to detected NPC | Median of calibrated transition probability matrix (Supplementary Fig 5) | Quartile 1 | Quartile 3 |  |  |
| Two Anti-EBV IgA Screening |  |  |  |  | ^16^ |
| Sensitivity | 0.903 | 0.853 | 0.953 | binomial |  |
| Specificity | 0.962 | 0.942 | 0.982 | binomial |  |
| Compliance | 1.000 | 0.700 | 1.000 |  |  |
| Proportion of Subjects with High Risk | Estimated in general population and NPC patients respectively (Supplementary Fig 4) |  |  |  |  |
| Initial Stage Distribution of Undetected NPC |  |  |  |  | ^1^ |
| Stage I | 0.349 | 0.299 | 0.392 | Dirichlet |  |
| Stage II | 0.381 | 0.334 | 0.433 | Dirichlet |  |
| Stage III | 0.230 | 0.191 | 0.281 | Dirichlet |  |
| Stage IVA/B | 0.040 | 0.025 | 0.069 | Dirichlet |  |
| Stage IVC | 0.000 | 0.000 | 0.000 | Dirichlet |  |
| Stage-Specific Detected NPC to recurrence and death | Time-dependent transition probability from NPC-specific database (Supplementary Table 6) |  |  |  |  |
| Cost (¥) |  |  |  |  |  |
| PRS SNP genotyping (per sample) | 120.00 | 60.00 | 480.00 | gamma | ^1,10^ |
| Two anti-EBV IgA serology test (per sample) | 108.61 | 54.31 | 217.22 | gamma | ^1^ |
| Nasopharyngoscopy | 194.30 | 97.15 | 388.60 | gamma | ^11^ |
| Costs of diagnosis and treatment |  |  |  |  | ^11,17,18^ |
| Stage I | 82 733.48 | 41 366.74 | 165 466.96 | gamma |  |
| Stage II | 94 040.13 | 47 020.07 | 188 080.26 | gamma |  |
| Stage III/IVA-B | 114 422.80 | 57 211.40 | 228 845.60 | gamma |  |
| Stage IVC | 79 332.42 | 39 666.21 | 158 664.84 | gamma |  |
| Cost of each follow-up visit | 1693.22 | 846.61 | 3386.44 | gamma | ^19^ |
| Total cost of diagnosis and re-treatment |  |  |  |  | ^11,19^ |
| Local recurrence | 105 572.97 | 52 786.49 | 211 145.94 | gamma |  |
| Regional recurrence | 90 180.39 | 45 090.20 | 180 360.78 | gamma |  |
| Distant metastasis | 209 600.46 | 104 800.23 | 419 200.92 | gamma |  |
| Health state utilities |  |  |  |  | ^20^ |
| Perfect health | 1.000 | — | — |  |  |
| Recurrence (LR, RR and DM), or *de novo* metastatic disease | 0.620 | 0.570 | 0.670 | beta |  |
| Under treatment with IMRT alone [months 1-3] | 0.884 | 0.834 | 0.934 | beta |  |
| Under treatment with IMRT and chemotherapy [months 1-3] | 0.833 | 0.783 | 0.883 | beta |  |
| Remission after IMRT alone [months 4-12] | 0.895 | 0.845 | 0.945 | beta |  |
| Remission after IMRT and chemotherapy [months 4-12] | 0.847 | 0.797 | 0.897 | beta |  |
| Remission after IMRT alone [months 12-24] | 0.911 | 0.861 | 0.961 | beta |  |
| Remission after IMRT and chemotherapy [months 12-24] | 0.863 | 0.813 | 0.913 | beta |  |
| Remission after IMRT alone [months 24-96] | 0.881 | 0.831 | 0.931 | beta |  |
| Remission after IMRT and chemotherapy [months 24-96] | 0.833 | 0.783 | 0.883 | beta |  |
| Remission after IMRT alone [months 96+] | 0.793 | 0.743 | 0.843 | beta |  |
| Remission after IMRT and chemotherapy [months 96+] | 0.745 | 0.695 | 0.795 | beta |  |
| Death | 0.000 |  |  |  |  |
| Discount Rate | 0.030 | 0.000 | 0.050 | beta | ^1,20^ |

**Supplementary Table 2. Performance characteristics for polygenic risk-stratified NPC screening strategies in individuals aged 50.**

| Strategy | Sensitivity | Specificity | PPV |
| --- | --- | --- | --- |
| Age-based strategy^a^ | 90.3% | 96.2% | 4.8% |
| Polygenic risk-stratified strategy^b^ |  |  |  |
| Male (10-year AR) |  |  |  |
| 0.30% | 87.6% | 96.4% | 4.9% |
| 0.40% | 83.4% | 96.8% | 5.3% |
| 0.50% | 78.1% | 97.3% | 5.8% |
| 0.60% | 70.9% | 97.7% | 6.2% |
| 0.70% | 63.9% | 98.2% | 7.0% |
| 0.80% | 56.6% | 98.5% | 7.3% |
| 0.90% | 49.2% | 98.9% | 8.6% |
| 1.00% | 42.1% | 99.1% | 9.2% |
| Female (10-year AR) |  |  |  |
| 0.10% | 84.9% | 96.7% | 5.2% |
| 0.15% | 75.1% | 97.5% | 5.9% |
| 0.20% | 62.2% | 98.3% | 7.2% |
| 0.25% | 48.6% | 99.0% | 9.2% |
| 0.30% | 36.3% | 99.3% | 9.3% |

^a^ the age-based strategy utilized two anti-EBV IgA antibodies, and its performance has been reported in the study by Ji et al^16^.

^b^ the performance of polygenic risk-stratified strategies was based on the performance of the age-based strategy and the proportion of high-risk individuals in the general population and NPC patients.

**Supplementary Table 3. Age-specific background mortality rate.**

| Age Group | Both sexes | Male | Female |
| --- | --- | --- | --- |
| 20-24 | 5.63×10^-4^ | 7.92×10^-4^ | 3.05×10^-4^ |
| 25-29 | 6.11×10^-4^ | 8.75×10^-4^ | 3.22×10^-4^ |
| 30-34 | 8.50×10^-4^ | 1.22×10^-3^ | 4.52×10^-4^ |
| 35-39 | 1.24×10^-3^ | 1.77×10^-3^ | 6.83×10^-4^ |
| 40-44 | 1.87×10^-3^ | 2.63×10^-3^ | 1.08×10^-3^ |
| 45-49 | 2.48×10^-3^ | 3.42×10^-3^ | 1.51×10^-3^ |
| 50-54 | 3.95×10^-3^ | 5.33×10^-3^ | 2.55×10^-3^ |
| 55-59 | 6.19×10^-3^ | 8.23×10^-3^ | 4.11×10^-3^ |
| 60-64 | 1.00×10^-2^ | 1.31×10^-2^ | 6.89×10^-3^ |
| 65-69 | 1.63×10^-2^ | 2.09×10^-2^ | 1.18×10^-2^ |
| 70-74 | 2.95×10^-2^ | 3.76×10^-2^ | 2.20×10^-2^ |
| 75-79 | 4.98×10^-2^ | 6.30×10^-2^ | 3.84×10^-2^ |
| 80-84 | 9.12×10^-2^ | 1.15×10^-1^ | 7.38×10^-2^ |
| 85-89 | 2.00×10^-1^ | 2.65×10^-1^ | 1.63×10^-1^ |

Note: Age-specific background mortality rate were generated from the China life table ^2^.

**Supplementary Table 4. Stage- and age-specific incidences rates of NPC for Asian and Pacific Islander from the SEER-18 dataset and its proportions of each stage in different age groups.**

| Age groups | Incidence rate of NPC (per 100 000) | Stage I | Stage II | Stage III | Stage IVA-B | Stage IVC |
| --- | --- | --- | --- | --- | --- | --- |
| 20-24 | 0.27 | 0.01 (4.89%) | 0.04 (14.29%) | 0.11 (42.86%) | 0.08 (28.57%) | 0.03 (9.40%) |
| 25-29 | 0.61 | 0.05 (8.16%) | 0.10 (16.31%) | 0.16 (26.59%) | 0.20 (32.63%) | 0.10 (16.31%) |
| 30-34 | 1.27 | 0.09 (7.32%) | 0.25 (19.28%) | 0.35 (27.54%) | 0.42 (33.04%) | 0.16 (12.82%) |
| 35-39 | 2.37 | 0.16 (6.91%) | 0.42 (17.73%) | 0.74 (31.03%) | 0.77 (32.51%) | 0.28 (11.83%) |
| 40-44 | 3.46 | 0.19 (5.54%) | 0.78 (22.49%) | 0.93 (26.94%) | 1.15 (33.21%) | 0.41 (11.81%) |
| 45-49 | 4.44 | 0.43 (9.70%) | 1.14 (25.60%) | 1.10 (24.72%) | 1.28 (28.82%) | 0.50 (11.17%) |
| 50-54 | 5.02 | 0.39 (7.85%) | 1.29 (25.77%) | 1.28 (25.49%) | 1.53 (30.53%) | 0.52 (10.36%) |
| 55-59 | 5.80 | 0.62 (10.65%) | 1.37 (23.53%) | 1.30 (22.41%) | 1.89 (32.49%) | 0.63 (10.93%) |
| 60-64 | 5.43 | 0.54 (9.95%) | 0.92 (16.87%) | 1.35 (24.90%) | 1.98 (36.41%) | 0.64 (11.87%) |
| 65-69 | 5.41 | 0.70 (12.97%) | 1.29 (23.78%) | 1.58 (29.19%) | 1.38 (25.41%) | 0.47 (8.65%) |
| 70-74 | 5.94 | 0.73 (12.24%) | 1.34 (22.45%) | 1.34 (22.45%) | 1.62 (27.21%) | 0.93 (15.64%) |
| 75-79 | 4.76 | 0.66 (13.75%) | 1.37 (28.76%) | 1.07 (22.50%) | 1.13 (23.74%) | 0.54 (11.25%) |
| 80-84 | 4.52 | 0.74 (16.36%) | 0.90 (20.01%) | 0.82 (18.18%) | 1.23 (27.27%) | 0.82 (18.18%) |
| 85-89 | 3.34 | 0.37 (11.12%) | 0.50 (14.80%) | 0.74 (22.22%) | 0.99 (29.64%) | 0.74 (22.22%) |

**Supplementary Table 5. The characteristics of non-metastatic NPC in Sun Yat-sen University Cancer Center.**

Continue

| Characteristics | No. of Patients (%) |  | Family history of NPC |  |
| --- | --- | --- | --- | --- |
|  |  |  | No | 7444 (73.5) |
|  | (N= 10,126) |  | Yes | 2682 (26.5) |
| Age, years |  |  | Clinical stage^b^ |  |
| <30 | 848 (8.4) |  | I | 328 (3.2) |
| 30-34 | 910 (9.0) |  | II | 1393 (13.8) |
| 35-39 | 1439 (14.2) |  | III | 5011 (49.5) |
| 40-44 | 1771 (17.5) |  | IVA-B | 3394 (33.5) |
| 45-49 | 1721 (17.0) |  | T stage |  |
| 50-54 | 1255 (12.4) |  | T1 | 965 ( 9.5) |
| 55-59 | 1039 (10.3) |  | T2 | 1780 (17.6) |
| 60-64 | 651 (6.4) |  | T3 | 4888 (48.3) |
| 65-69 | 315 (3.1) |  | T4 | 2493 (24.6) |
| ≥70 | 177 (1.7) |  | N stage |  |
| Sex |  |  | N0 | 1236 (12.2) |
| Male | 7440 (73.5) |  | N1 | 4482 (44.3) |
| Female | 2686 (26.5) |  | N2 | 3239 (32.0) |
| Histology, WHO type^a^ |  |  | N3 | 1169 (11.5) |
| I | 66 (0.7) |  | Induction chemotherapy |  |
| II | 205 (2.0) |  | No | 5062 (50.0) |
| III | 9855 (97.3) |  | Yes | 5064 (50.0) |
| Smoking |  |  | Concurrent chemotherapy |  |
| No | 6575 (64.9) |  | No | 2234 (22.1) |
| Yes | 3551 (35.1) |  | Yes | 7892 (77.9) |
| Alcohol |  |  | Adjuvant chemotherapy |  |
| No | 8732 (86.2) |  | No | 9451 (94.6) |
| Yes | 1394 (13.8) |  | Yes | 543 (5.4) |

^a^ WHO Type I refers to the keratinizing squamous cell carcinoma; WHO Type II refers to the differentiated non-keratinizing carcinoma; WHO Type III refers to the undifferentiated non-keratinizing carcinoma.

^b^ According to the 7th edition of AJCC/UICC Staging System

**Supplementary Table 6. The parameters of estimating recurrence and survival for NPC.**

| **Parameter** | **Base-Case Estimate** | **Range** | **Reference** |
| --- | --- | --- | --- |
| Local relapse |  |  | NPC-specific database ^5^ |
| Stage I | 3.9% by 8 years | 2.0%–8.0% |  |
| Log-normal model parameters | μ=7.945, σ=1.811 |  |  |
| Stage II | 5.5% by 8 years | 4.3%–7.2% |  |
| Log-normal model parameters | μ=8.197, σ=2.254 |  |  |
| Stage III | 8.1% by 8 years | 7.3%–9.0% |  |
| Log-normal model parameters | μ=7.432, σ=2.047 |  |  |
| Stage IVA-B | 15.0% by 8 years | 13.5%–16.6% |  |
| Log-normal model parameters | μ=6.557, σ=1.921 |  |  |
| Regional relapse |  |  | NPC-specific database ^5^ |
| Stage I | 4.2% by 8 years | 0.8%–17.7% |  |
| Log-normal model parameters | μ=6.213, σ=0.790 |  |  |
| Stage II | 4.7% by 8 years | 3.7%–6.5% |  |
| Log-normal model parameters | μ=9.216, σ=2.747 |  |  |
| Stage III | 7.2% by 8 years | 6.5%–8.3% |  |
| Log-normal model parameters | μ=7.928, σ=2.302 |  |  |
| Stage IVA-B | 10.2% by 8 years | 9.0%–11.5% |  |
| Log-normal model parameters | μ=7.526, σ=2.322 |  |  |
| Distant metastasis |  |  | NPC-specific database ^5^ |
| Stage I | 3.2% by 8 years | 1.5%–6.0% |  |
| Gompertz model parameters | λ=0.0009, γ=-0.0304 |  |  |
| Stage II | 5.8% by 8 years | 4.5%–7.3% |  |
| Gompertz model parameters | λ=0.0015, γ=-0.0223 |  |  |
| Stage III | 12.0% by 8 years | 11.0%–13.0% |  |
| Gompertz model parameters | λ=0.0036, γ=-0.0261 |  |  |
| Stage IVA-B | 23.2% by 8 years | 21.6%–24.7% |  |
| Gompertz model parameters | λ=0.0080, γ=-0.0285 |  |  |
| Death |  |  | NPC-specific database ^5^ |
| Stage I^a^ | 0.0% during follow-up | 0.0%-0.0% |  |
| Stage II^a^ | 2.8% by 8 years | 1.8%-4.6% |  |
| Gompertz model parameters | λ=6.15×10^-5^, γ=0.0026 |  |  |
| Stage III^a^ | 3.2% by 8 years | 2.7%-3.9% |  |
| Gompertz model parameters | λ=1.96×10^-4^, γ=0.0118 |  |  |
| Stage IVA-B^a^ | 6.4% by 8 years | 5.4%-7.6% |  |
| Gompertz model parameters | λ=4.02×10^-4^, γ=0.0118 |  |  |
| Stage IVC | 86.9% by 5 years | 82.5%-91.2% | ^8^ |
| Log-normal model parameters | μ=3.138, σ=0.851 |  |  |
| Probability of death of disease recurrence after treatment |  |  |  |
| local relapse | 0.0176  (65.5% by 5 years) | 0.0160–0.0213 | ^21-23^ |
| regional relapse | 0.0155  (60.9% by 5 years) | 0.0117–0.0273 | ^24,25^ |
| distant metastasis | 0.0458  (94.0% by 5 years) | 0.0422–0.0516 | ^26,27^ |

^a^ Death resulting from causes other than the recurrence (LR, RR and DM) of NPC.

**Supplementary Table 7. Model validation of the Markov model-predicted survival compared with the real-world observed survival for non-metastatic NPC.**

| Outcomes | Years | Stages | Actual | Model (SD)^a^ | Absolute Difference |
| --- | --- | --- | --- | --- | --- |
| LR | 1 year | Total | 99.20% |  |  |
|  |  | Stage I | 99.70% | 99.80% (0.32%) | 0.10% |
|  |  | Stage II | 99.60% | 99.40% (0.18%) | 0.20% |
|  |  | Stage III | 99.40% | 99.20% (0.11%) | 0.20% |
|  |  | Stage IVA-B | 98.60% | 98.30% (0.19%) | 0.30% |
|  | 3 years | Total | 95.40% |  |  |
|  |  | Stage I | 99.40% | 99.00% (0.47%) | 0.40% |
|  |  | Stage II | 97.60% | 97.90% (0.34%) | 0.30% |
|  |  | Stage III | 96.40% | 97.00% (0.21%) | 0.60% |
|  |  | Stage IVA-B | 92.60% | 93.90% (0.36%) | 1.30% |
|  | 5 years | Total | 93.30% |  |  |
|  |  | Stage I | 98.10% | 97.90% (1.04%) | 0.20% |
|  |  | Stage II | 96.40% | 96.40% (0.50%) | 0.00% |
|  |  | Stage III | 94.50% | 94.80% (0.31%) | 0.30% |
|  |  | Stage IVA-B | 89.60% | 90.00% (0.50%) | 0.40% |
|  | 8 years | Total | 91.50% |  |  |
|  |  | Stage I | 96.90% | 96.10% (2.80%) | 0.80% |
|  |  | Stage II | 95.02% | 94.50% (0.75%) | 0.52% |
|  |  | Stage III | 92.90% | 91.90% (0.47%) | 1.00% |
|  |  | Stage IVA-B | 87.00% | 85.00% (0.78%) | 2.00% |
| RR | 1 year | Total | 99.10% |  |  |
|  |  | Stage I | 100.00% | 100.00% (0.04%) | 0.00% |
|  |  | Stage II | 99.40% | 99.20% (0.20%) | 0.20% |
|  |  | Stage III | 99.20% | 99.10% (0.12%) | 0.10% |
|  |  | Stage IVA-B | 98.80% | 98.50% (0.18%) | 0.30% |
|  | 3 years | Total | 96.00% |  |  |
|  |  | Stage I | 99.70% | 99.80% (0.30%) | 0.10% |
|  |  | Stage II | 97.50% | 97.90% (0.35%) | 0.40% |
|  |  | Stage III | 96.40% | 97.00% (0.21%) | 0.60% |
|  |  | Stage IVA-B | 94.40% | 95.50% (0.31%) | 1.10% |
|  | 5 years | Total | 94.50% |  |  |
|  |  | Stage I | 99.70% | 98.90% (4.16%) | 0.80% |
|  |  | Stage II | 96.50% | 96.80% (0.47%) | 0.30% |
|  |  | Stage III | 94.80% | 95.20% (0.30%) | 0.40% |
|  |  | Stage IVA-B | 92.60% | 93.00% (0.42%) | 0.40% |
|  | 8 years | Total | 93.50% |  |  |
|  |  | Stage I | 98.02% | 95.70% (8.19%) | 2.22% |
|  |  | Stage II | 96.10% | 95.30% (0.72%) | 0.80% |
|  |  | Stage III | 94.00% | 92.80% (0.46%) | 1.20% |
|  |  | Stage IVA-B | 91.30% | 89.80% (0.64%) | 1.50% |
| DM | 1 year | Total | 95.90% |  |  |
|  |  | Stage I | 99.70% | 99.00% (0.48%) | 0.70% |
|  |  | Stage II | 98.90% | 98.40% (0.25%) | 0.50% |
|  |  | Stage III | 96.80% | 96.30% (0.22%) | 0.50% |
|  |  | Stage IVA-B | 93.00% | 92.20% (0.38%) | 0.80% |
|  | 3 years | Total | 89.40% |  |  |
|  |  | Stage I | 97.90% | 97.80% (0.82%) | 0.10% |
|  |  | Stage II | 96.30% | 96.40% (0.48%) | 0.10% |
|  |  | Stage III | 91.70% | 91.90% (0.36%) | 0.20% |
|  |  | Stage IVA-B | 82.50% | 83.40% (0.60%) | 0.90% |
|  | 5 years | Total | 87.10% |  |  |
|  |  | Stage I | 97.20% | 97.20% (1.00%) | 0.00% |
|  |  | Stage II | 95.00% | 95.20% (0.54%) | 0.20% |
|  |  | Stage III | 89.60% | 89.60% (0.42%) | 0.00% |
|  |  | Stage IVA-B | 79.10% | 79.40% (0.69%) | 0.30% |
|  | 8 years | Total | 85.70% |  |  |
|  |  | Stage I | 97.20% | 96.80% (1.16%) | 0.40% |
|  |  | Stage II | 94.40% | 94.20% (0.68%) | 0.20% |
|  |  | Stage III | 88.10% | 88.00% (0.49%) | 0.10% |
|  |  | Stage IVA-B | 76.90% | 76.80% (0.78%) | 0.10% |

^a^ The estimated survival rates were obtained through 1000 simulation replications.

**Supplementary Table 8. The costs of items in screening, diagnosis and treatment of NPC and follow-up care.**

| Item (China) | Cost (¥) | Range (¥) | Reference |
| --- | --- | --- | --- |
| **Screening** |  |  |  |
| Total costs of PRS (per sample) | 120.00 | 60.00-480.00 |  |
| Reagents and consumables | 55.00 | 27.50-110.00 | ^10^ |
| Wastage of reagents ^a^ | 16.50 | 8.25-33.00 | Expert evaluation |
| Transport Cost | 0.87 | 0.44-1.74 | ^1^ |
| Phlebotomist Costs | 7.10 | 3.55-14.20 | ^1^ |
| Admin. Assistant Costs | 4.62 | 2.31-9.24 | ^1^ |
| Laboratory Technician Costs: Automated DNA extraction | 4.89 | 2.45-9.78 | ^1^ |
| Laboratory Technician Costs: Automated genotyping | 6.77 | 3.39-13.54 | ^1^ |
| Statistical analyses Costs: Data quality control, storage, analysis, and risk assessment | 15.60 | 7.80-31.20 | Expert evaluation |
| Overhead | 8.65 | 4.33-17.30 | Expert evaluation |
| Total costs of testing anti-EBV IgA  (per sample) | 108.61 | 54.31-217.22 | ^1^ |
| Nasopharyngoscopy | 194.30 | 97.15-388.60 | ^11^ |
| **Diagnosis and treatment** |  |  |  |
| Costs of diagnosis |  |  | ^11^ |
| Routine clinic visit | 473.29 | 236.65-946.58 |  |
| Complete blood count | 18.76 | 9.38-37.52 |  |
| Comprehensive metabolic panel | 260.23 | 130.12-520.46 |  |
| Nasopharyngoscopy | 194.30 | 97.15-388.60 |  |
| Plasma cfEBV DNA load test | 147.80 | 73.90-295.60 |  |
| MRI scan with contrast (head and neck) | 2232.57 | 1116.29-4465.14 |  |
| CT scan with contrast | 1228.38 | 614.19-2456.76 |  |
| CT chest | 614.19 | 307.10-1228.38 |  |
| CT abdomen | 614.19 | 307.10-1228.38 |  |
| Bone scintigraphy | 841.86 | 420.93-1683.72 |  |
| PET/CT scan | 8872.07 | 4436.04-17744.14 |  |
| Work-up for pathological diagnosis | 1013.24 | 506.62-2026.48 | ^11^ |
| Pre-procedure labs | 514.23 | 257.12-1028.46 |  |
| Nasopharyngoscopy-guided biopsy | 298.42 | 149.21-596.84 |  |
| Pathology processing | 200.60 | 100.30-401.20 |  |
| Total cost of IMRT | 64 830.47 | 32 415.24-129 660.94 | ^18^ |
| Costs of chemotherapy (per cycle) |  |  | ^17^ |
| Gemcitabine | 3836.89 | 1918.45-7673.78 |  |
| Cisplatin^b^ | 92.39 | 46.20-184.78 |  |
| Hydration | 364.68 | 182.34-729.36 |  |
| Antiemetic drugs | 567.89 | 283.95-1135.78 |  |
| Hospitalization | 792.21 | 396.11-1584.42 |  |
| PICC/one time | 1617.31 | 808.66-3234.62 |  |
| Other |  |  | ^17^ |
| Subsequent treatment | 3714.08 | 1857.04-7428.16 |  |
| Best supportive care | 360.39 | 180.20-720.78 |  |
| Work-up for imaging detected local relapse | 1013.24 | 506.62-2026.48 | ^11^ |
| Pre-procedure labs | 514.23 | 257.12-1028.46 |  |
| Nasopharyngoscopy-guided biopsy | 298.42 | 149.21-596.84 |  |
| Pathology processing | 200.60 | 100.30-401.20 |  |
| Work-up for imaging detected regional relapse | 1018.80 | 509.40-2037.60 | ^11^ |
| Pre-procedure labs | 514.23 | 257.12-1028.46 |  |
| CT-guided biopsy | 303.98 | 151.99-607.96 |  |
| Pathology processing | 200.60 | 100.30-401.20 |  |
| Work-up for imaging detected distant metastasis | 1104.83 | 552.42-2209.66 | ^11^ |
| Pre-procedure labs | 514.23 | 257.12-1028.46 |  |
| CT-guided biopsy | 390.01 | 195.01-780.02 |  |
| Pathology processing | 200.60 | 100.30-401.20 |  |
| Total cost of re-treatment during the treatment period |  |  | ^19^ |
| Local recurrence | 88 531.12 | 44 265.56-177 062.24 |  |
| Regional recurrence | 73 132.98 | 36 566.49-146 265.96 |  |
| Distant metastasis | 192 467.01 | 96 233.51-384 934.02 |  |
| **Follow-up care^c^** |  |  |  |
| Cost of each follow-up visit | 1693.22 | 846.61-3386.44 | ^19^ |

^a^ Taking into account the potential need for retesting failed samples and conducting quality control by retesting some samples to ensure accurate testing, we have set a wastage ratio of 30%.

^b^ Based on the mean Asian body surface area of 1.72 m^2^.

^c^ A follow-up period of 5 years after the treatment.

**Supplementary Table 9. Base-case analysis of only age-based screening strategies in female population.**

| Starting age | Screening strategy^a^ | Incremental costs^b^ | Incremental QALYs^b^ | ICER^c^ | |
| --- | --- | --- | --- | --- | --- |
|  |  |  |  | *vs.* no screening | *vs.* preceding strategy on efficiency frontier |
| 30-34 | Age-5 | 55 677 744 | 309 | 179 987 | 179 987 |
|  | Age-4 | 67 782 459 | 355 | 191 176 | 267 726 |
|  | Age-3 | 88 751 403 | 410 | 216 605 | 379 996 |
|  | Age-2 | 130 407 592 | 477 | 273 272 | 617 403 |
|  | Age-1 | 257 298 023 | 564 | 456 059 | 1 459 031 |
| 35-39 | Age-5 | 51 465 363 | 312 | 165 186 | 165 186 |
|  | Age-4 | 62 661 259 | 354 | 177 159 | 265 683 |
|  | Age-3 | 82 034 628 | 406 | 202 304 | 374 003 |
|  | Age-2 | 120 288 485 | 468 | 256 833 | 608 634 |
|  | Age-1 | 236 363 134 | 550 | 430 066 | 1 428 717 |
| 40-44 | Age-5 | 46 797 195 | 278 | 168 616 | 168 616 |
|  | Age-4 | 57 023 950 | 314 | 181 667 | 281 295 |
|  | Age-3 | 73 684 150 | 358 | 206 054 | 381 205 |
|  | Age-2 | 108 141 869 | 412 | 262 668 | 636 820 |
|  | Age-1 | 212 742 015 | 482 | 441 460 | 1 490 031 |
| 45-49 | Age-5 | 41 488 848 | 235 | 176 546 | 176 546 |
|  | Age-4 | 50 689 654 | 263 | 192 483 | 324 624 |
|  | Age-3 | 65 100 038 | 297 | 219 287 | 429 840 |
|  | Age-2 | 95 316 733 | 339 | 281 053 | 714 850 |
|  | Age-1 | 185 867 080 | 394 | 471 807 | 1 652 198 |
| 50-54 | Age-5 | 35 483 388 | 175 | 203 150 | 203 150 |
|  | Age-4 | 42 316 404 | 193 | 219 114 | 370 173 |
|  | Age-3 | 55 506 376 | 217 | 255 222 | 541 505 |
|  | Age-2 | 79 613 424 | 246 | 323 516 | 842 756 |
|  | Age-1 | 155 230 322 | 285 | 544 488 | 1 938 597 |
| 55-59 | Age-5 | 28 675 039 | 109 | 264 250 | 264 250 |
|  | Age-4 | 34 012 324 | 120 | 283 954 | 473 752 |
|  | Age-3 | 43 186 269 | 134 | 322 599 | 651 142 |
|  | Age-2 | 63 015 114 | 153 | 413 208 | 1 064 236 |
|  | Age-1 | 120 257 366 | 177 | 680 643 | 2 367 339 |
| 60-64 | Age-5 | 20 751 525 | 63 | 329 871 | 329 871 |
|  | Age-4 | 24 467 363 | 68 | 357 700 | 676 345 |
|  | Age-3 | 30 333 743 | 75 | 405 012 | 903 354 |
|  | Age-2 | 42 251 586 | 84 | 505 408 | 1 369 395 |
|  | Age-1 | 79 915 015 | 96 | 828 736 | 2 935 346 |
| 65-69 | Age-5 | 11 493 564 | 23 | 497 811 | 497 811 |
|  | Age-4 | 13 448 988 | 25 | 534 755 | 948 498 |
|  | Age-3 | 15 551 992 | 27 | 576 047 | 1 137 989 |
|  | Age-2 | 19 774 135 | 30 | 668 913 | 1 646 830 |
|  | Age-1 | 32 664 958 | 34 | 972 614 | 3 204 201 |

^a^ The screening strategies are labeled as follows: for age-based strategies, Age-screening frequency;

^b^ Incremental health benefits gained, and costs incurred from age-based screening strategies relative to the no-screening strategy per 100 000 women with no prior NPC diagnosis, and all health utility and costs were discount at a 3% annual rate.

^c^ Their respective ICERs based on the average values of each age

**Supplementary Table 10. Base-case analysis of age-based and** **polygenic risk-stratified screening strategies in male population aged 55-69 years.**

| Starting age | Screening strategy^a^ | Incremental costs^b^ | Incremental QALYs^b^ | ICER^c^ | |
| --- | --- | --- | --- | --- | --- |
|  |  |  |  | *vs.* no screening | *vs.* preceding strategy on efficiency frontier |
| 55-59 | Age-5 | 27 382 976 | 365 | 75 055 | 75 055 |
|  | Age-4 | 32 351 879 | 401 | 80 693 | 137 697 |
|  | Age-3 | 40 928 040 | 447 | 91 579 | 186 489 |
|  | Age-2 | 59 552 813 | 508 | 117 149 | 303 160 |
|  | AR-0.4%-1 | 77 058 143 | 537 | 143 450 | 607 214 |
|  | Age-1 | 114 013 919 | 586 | 194 440 | 751 272 |
| 60-64 | Age-5 | 20 413 038 | 202 | 101 070 | 101 070 |
|  | Age-4 | 23 935 730 | 221 | 108 071 | 180 529 |
|  | Age-3 | 29 513 900 | 244 | 120 741 | 242 984 |
|  | Age-2 | 40 875 553 | 275 | 148 604 | 371 001 |
|  | Age-1 | 77 052 913 | 320 | 240 950 | 808 898 |
| 65-69 | Age-5 | 11 836 613 | 95 | 124 865 | 124 865 |
|  | Age-4 | 13 777 888 | 103 | 133 174 | 224 113 |
|  | Age-3 | 15 846 782 | 111 | 142 886 | 277 805 |
|  | Age-2 | 19 979 588 | 121 | 164 705 | 397 379 |
|  | Age-1 | 32 600 377 | 137 | 237 569 | 792 777 |

^a^ The screening strategies are labeled as follows: for age-based strategies, “Age-screening frequency”; for polygenic risk-stratified strategies, “AR-10-year NPC absolute risk threshold-screening frequency”.

^b^ Incremental health benefits gained, and costs incurred from age-based screening strategies relative to the no-screening strategy per 100 000 men with no prior NPC diagnosis, and all health utililty and costs were discount at a 3% annual rate.

^c^ Their respective ICERs based on the average values of each age.

**Supplementary Table 11. Screening utilization and outcomes using the screening strategies on cost-effectiveness efficiency frontiers in male population aged 55-69 years.**

| Starting age | Screening strategy^a^ | Anti-EBV IgA tests, n | Nasal endoscopy tests, n | Detected NPC, n^b^ | | | | NPC deaths averted |
| --- | --- | --- | --- | --- | --- | --- | --- | --- |
|  |  |  |  | Early stage NPC | | Locoregionally advanced NPC | Recurrent NPC |  |
| 55-59 | Base | — | — | 215 | 347 | | 207 | — |
|  | Age-5 | 280 931 | 10 882 | 408 | 219 | | 132 | 71 |
|  | Age-4 | 336 056 | 12 998 | 434 | 200 | | 123 | 79 |
|  | Age-3 | 428 623 | 16 542 | 464 | 176 | | 114 | 89 |
|  | Age-2 | 629 921 | 24 228 | 508 | 142 | | 101 | 102 |
|  | AR-0.4%-1 | 672 409 | 25 814 | 513 | 136 | | 99 | 103 |
|  | Age-1 | 1 201 275 | 45 986 | 550 | 108 | | 88 | 114 |
| 60-64 | Base | — | — | 152 | 249 | | 145 | — |
|  | Age-5 | 191 251 | 7 404 | 280 | 168 | | 97 | 46 |
|  | Age-4 | 227 853 | 8 810 | 297 | 156 | | 92 | 51 |
|  | Age-3 | 283 865 | 10 955 | 316 | 143 | | 86 | 57 |
|  | Age-2 | 475 978 | 18 270 | 352 | 114 | | 76 | 68 |
|  | Age-1 | 751 331 | 28 772 | 370 | 100 | | 70 | 73 |
| 65-69 | Base | — | — | 101 | 171 | | 96 | — |
|  | Age-5 | 99 664 | 3 866 | 174 | 128 | | 68 | 26 |
|  | Age-4 | 117 955 | 4 571 | 184 | 121 | | 66 | 29 |
|  | Age-3 | 137 034 | 5 303 | 192 | 117 | | 63 | 32 |
|  | Age-2 | 175 213 | 6 764 | 202 | 109 | | 61 | 35 |
|  | Age-1 | 290 550 | 11 161 | 216 | 100 | | 57 | 39 |

^a^ The screening strategies are labeled as follows: for age-based strategies, “Age-screening frequency”; for polygenic risk-stratified strategies, “AR-10-year NPC absolute risk threshold-screening frequency”.

^b^ Early stage NPC includes those in stage I and stage II; Locoregionally advanced NPC includes those in stage III and stage IVA-B; Recurrent NPC includes those who were *de novo* metastasis and those who developed local relapse, regional relapse or metastasis after treatment.

**Supplementary Table 12. Base-case analysis of age-based and polygenic risk-stratified screening strategies in female population.**

| Starting age | Screening strategy^a^ | Incremental costs^b^ | Incremental QALYs^b^ | ICER^c^ | |
| --- | --- | --- | --- | --- | --- |
|  |  |  |  | *vs.* no screening | *vs.* preceding strategy on efficiency frontier |
| 30-34 | AR-0.25%-2 | 32 278 597 | 244 | 132 533 | 132 533 |
|  | AR-0.30%-1 | 37 180 212 | 277 | 134 329 | 147 490 |
|  | AR-0.25%-1 | 52 958 570 | 350 | 151 409 | 216 185 |
|  | Age-5^d^ | 55 677 744 | 309 | 179 987 | Strongly dominated^e^ |
|  | AR-0.20%-1 | 84 100 679 | 422 | 199 252 | 430 668 |
|  | AR-0.15%-1 | 134 047 224 | 486 | 275 641 | 777 619 |
|  | AR-0.10%-1 | 198 460 026 | 535 | 371 153 | 1 330 847 |
|  | Age-1 | 257 298 023 | 564 | 456 059 | 1 996 846 |
| 35-39 | AR-0.30%-2 | 26 275 917 | 204 | 128 976 | 128 976 |
|  | AR-0.25%-2 | 34 898 224 | 270 | 129 358 | 130 538 |
|  | AR-0.30%-1 | 40 592 042 | 309 | 131 354 | 145 069 |
|  | Age-5^d^ | 51 465 363 | 312 | 165 185 | Weakly dominated^e^ |
|  | AR-0.25%-1 | 57 899 313 | 381 | 151 822 | 239 269 |
|  | AR-0.20%-1 | 91 125 966 | 445 | 204 608 | 519 123 |
|  | AR-0.15%-1 | 142 002 579 | 496 | 286 320 | 1 005 663 |
|  | AR-0.10%-1 | 200 369 629 | 531 | 377 597 | 1 682 714 |
|  | Age-1 | 236 363 134 | 550 | 430 066 | 1 899 120 |
| 40-44 | AR-0.30%-2 | 26 737 486 | 199 | 134 310 | 134 310 |
|  | AR-0.25%-2 | 34 854 703 | 257 | 135 484 | 139 501 |
|  | AR-0.30%-1 | 41 106 987 | 295 | 139 398 | 166 151 |
|  | Age-5^d^ | 46 797 195 | 278 | 168 616 | Strongly dominated^e^ |
|  | AR-0.25%-1 | 57 049 356 | 356 | 160 471 | 262 983 |
|  | AR-0.20%-1 | 89 156 380 | 406 | 219 469 | 632 954 |
|  | AR-0.15%-1 | 135 607 561 | 444 | 305 159 | 1 217 695 |
|  | AR-0.10%-1 | 185 906 003 | 469 | 396 079 | 2 013 387 |
|  | Age-1 | 212 742 015 | 482 | 441 460 | 2 140 104 |
| 45-49 | AR-0.25%-2 | 31 452 260 | 211 | 149 068 | 149 068 |
|  | AR-0.30%-1 | 36 105 147 | 238 | 151 578 | 171 050 |
|  | Age-5^d^ | 41 488 848 | 235 | 176 546 | Strongly dominated^e^ |
|  | AR-0.25%-1 | 49 731 772 | 289 | 172 347 | 270 575 |
|  | AR-0.20%-1 | 76 919 060 | 331 | 232 449 | 641 944 |
|  | AR-0.15%-1 | 117 373 803 | 363 | 323 525 | 1 268 609 |
|  | AR-0.10%-1 | 162 206 254 | 383 | 422 974 | 2 166 507 |
|  | Age-1 | 185 867 080 | 394 | 471 807 | 2 262 713 |
| 50-54 | AR-0.30%-1 | 28 394 541 | 159 | 178 223 | 178 223 |
|  | Age-5^d^ | 35 483 388 | 175 | 203 151 | Weakly dominated^e^ |
|  | AR-0.25%-1 | 39 935 785 | 199 | 200 934 | 292 699 |
|  | AR-0.20%-1 | 60 297 328 | 233 | 258 841 | 595 347 |
|  | AR-0.15%-1 | 93 880 349 | 259 | 362 536 | 1 291 493 |
|  | AR-0.10%-1 | 133 825 383 | 276 | 484 537 | 2 317 346 |
|  | Age-1 | 155 230 322 | 285 | 544 489 | 2 404 630 |
| 55-59 | AR-0.30%-1 | 20 728 754 | 82 | 251 933 | 251 933 |
|  | Age-5^d^ | 28 675 039 | 109 | 264 251 | 302 881 |
|  | Age-4 | 34 012 324 | 120 | 283 956 | 473 752 |
|  | AR-0.20%-1 | 42 705 225 | 136 | 314 814 | 547 694 |
|  | Age-2 | 63 015 114 | 153 | 413 208 | 1 205 348 |
|  | AR-0.15%-1 | 68 311 242 | 156 | 439 198 | 1 745 401 |
|  | AR-0.10%-1 | 102 014 977 | 169 | 602 450 | 2 442 823 |
|  | Age-1 | 120 257 366 | 177 | 680 642 | 2 482 393 |
| 60-64 | Age-5 | 20 751 525 | 63 | 329 875 | 329 875 |
|  | Age-4 | 24 467 363 | 68 | 357 703 | 676 329 |
|  | Age-3 | 30 333 743 | 75 | 405 014 | 903 335 |
|  | Age-2 | 42 251 586 | 84 | 505 413 | 1 369 451 |
|  | Age-1 | 79 915 015 | 96 | 828 736 | 2 935 157 |
| 65-69 | Age-5 | 11 493 564 | 23 | 497 810 | 497 810 |
|  | Age-4 | 13 448 988 | 25 | 534 755 | 948 503 |
|  | Age-3 | 15 551 992 | 27 | 576 048 | 1 138 033 |
|  | Age-2 | 19 774 135 | 30 | 668 914 | 1 646 823 |
|  | Age-1 | 32 664 958 | 34 | 972 614 | 3 204 178 |

^a^ The screening strategies are labeled as follows: for age-based strategies, “Age-screening frequency”; for polygenic risk-stratified strategies, “AR-10-year NPC absolute risk threshold-screening frequency”.

^b^ Additional health benefits gained, and costs incurred from age-based screening strategies relative to the no-screening strategy per 100 000 women with no prior NPC diagnosis, and all health utility and costs were discount at a 3% annual rate.

^c^ Their respective ICERs based on the average values of each age.

^d^ The optimal age-based strategy was included for ease of comparison.

^e^ A strongly dominated strategy was defined as a strategy for which another strategy existed that yielded better health benefit at lower cost, and a weakly dominated strategy was defined as a strategy dominated by a linear combination of 2 other strategies.

**Supplementary Table 13. Screening utilization and outcomes using the screening strategies on cost-effectiveness efficiency frontiers in female population.**

| Starting age | Screening strategy^a^ | Anti-EBV IgA tests, n | Nasal endoscopy tests, n | Detected NPC, n^b^ | | | NPC deaths averted |
| --- | --- | --- | --- | --- | --- | --- | --- |
|  |  |  |  | Early stage NPC | Locoregionally advanced NPC | Recurrent NPC |  |
| 30-34 | Base | — | — | 174 | 258 | 158 | — |
|  | AR-0.25%-2 | 317 307 | 12 141 | 298 | 165 | 107 | 46 |
|  | AR-0.30%-1 | 392 068 | 14 989 | 317 | 151 | 101 | 52 |
|  | AR-0.25%-1 | 634 738 | 24 237 | 355 | 120 | 87 | 65 |
|  | Age-5^c^ | 772 596 | 29 516 | 323 | 150 | 97 | 56 |
|  | AR-0.20%-1 | 1 095 944 | 41 802 | 391 | 91 | 75 | 76 |
|  | AR-0.15%-1 | 1 828 350 | 69 680 | 420 | 66 | 65 | 86 |
|  | AR-0.10%-1 | 2 742 561 | 104 462 | 440 | 49 | 58 | 92 |
|  | Age-1 | 3 662 246 | 139 441 | 450 | 40 | 55 | 95 |
| 35-39 | Base | — | — | 163 | 245 | 150 | — |
|  | AR-0.30%-2 | 194 659 | 7 457 | 254 | 179 | 112 | 34 |
|  | AR-0.25%-2 | 312 859 | 11 971 | 286 | 154 | 100 | 45 |
|  | AR-0.30%-1 | 387 208 | 14 803 | 304 | 140 | 93 | 51 |
|  | Age-5^c^ | 674 669 | 25 787 | 303 | 144 | 92 | 53 |
|  | AR-0.25%-1 | 621 726 | 23 740 | 340 | 111 | 80 | 63 |
|  | AR-0.20%-1 | 1 059 569 | 40 415 | 372 | 85 | 70 | 73 |
|  | AR-0.15%-1 | 1 735 701 | 66 153 | 397 | 64 | 62 | 81 |
|  | AR-0.10%-1 | 2 520 646 | 96 018 | 412 | 50 | 56 | 86 |
|  | Age-1 | 3 172 576 | 120 816 | 421 | 43 | 53 | 89 |
| 40-44 | Base | — | — | 146 | 222 | 135 | — |
|  | AR-0.30%-2 | 179 188 | 6 865 | 229 | 161 | 101 | 31 |
|  | AR-0.25%-2 | 281 768 | 10 783 | 258 | 139 | 90 | 41 |
|  | AR-0.30%-1 | 352 854 | 13 491 | 274 | 127 | 84 | 46 |
|  | Age-5^c^ | 577 444 | 22 077 | 272 | 132 | 84 | 48 |
|  | AR-0.25%-1 | 553 670 | 21 144 | 306 | 101 | 73 | 57 |
|  | AR-0.20%-1 | 939 983 | 35 858 | 333 | 79 | 63 | 66 |
|  | AR-0.15%-1 | 1 516 646 | 57 811 | 355 | 61 | 56 | 73 |
|  | AR-0.10%-1 | 2 165 215 | 82 488 | 368 | 50 | 52 | 77 |
|  | Age-1 | 2 686 375 | 102 312 | 375 | 44 | 49 | 79 |
| 45-49 | Base | — | — | 126 | 196 | 118 | — |
|  | AR-0.25%-2 | 224 094 | 8 579 | 220 | 128 | 80 | 35 |
|  | AR-0.30%-1 | 272 155 | 10 410 | 231 | 118 | 76 | 38 |
|  | Age-5^c^ | 480 789 | 18 387 | 236 | 118 | 73 | 41 |
|  | AR-0.25%-1 | 433 869 | 16 575 | 259 | 97 | 66 | 48 |
|  | AR-0.20%-1 | 740 181 | 28 244 | 283 | 77 | 58 | 56 |
|  | AR-0.15%-1 | 1 211 335 | 46 182 | 303 | 61 | 51 | 62 |
|  | AR-0.10%-1 | 1 754 554 | 66 852 | 315 | 50 | 47 | 66 |
|  | Age-1 | 2 203 030 | 83 912 | 321 | 45 | 45 | 68 |
| 50-54 | Base | — | — | 104 | 163 | 98 | — |
|  | AR-0.30%-1 | 176 033 | 6 738 | 182 | 108 | 66 | 28 |
|  | Age-5^c^ | 384 870 | 14 720 | 193 | 100 | 61 | 33 |
|  | AR-0.25%-1 | 303 177 | 11 587 | 205 | 89 | 58 | 36 |
|  | AR-0.20%-1 | 520 898 | 19 884 | 226 | 72 | 50 | 43 |
|  | AR-0.15%-1 | 887 134 | 33 829 | 243 | 59 | 45 | 49 |
|  | AR-0.10%-1 | 1 336 266 | 50 920 | 254 | 49 | 41 | 52 |
|  | Age-1 | 1 723 155 | 65 638 | 260 | 44 | 39 | 54 |
| 55-59 | Base | — | — | 80 | 126 | 75 | — |
|  | AR-0.30%-1 | 90 444 | 3 464 | 127 | 93 | 55 | 17 |
|  | Age-5^c^ | 289 816 | 11 082 | 145 | 82 | 48 | 24 |
|  | Age-4 | 347 232 | 13 272 | 153 | 75 | 45 | 26 |
|  | AR-0.20%-1 | 315 090 | 12 032 | 164 | 65 | 42 | 29 |
|  | Age-2 | 653 280 | 24 924 | 180 | 54 | 37 | 34 |
|  | AR-0.15%-1 | 576 181 | 21 977 | 179 | 53 | 37 | 34 |
|  | AR-0.10%-1 | 926 530 | 35 310 | 189 | 46 | 34 | 37 |
|  | Age-1 | 1 247 412 | 47 517 | 194 | 41 | 32 | 39 |
| 60-64 | Base | — | — | 59 | 96 | 55 | — |
|  | Age-5 | 195 192 | 7 466 | 105 | 66 | 37 | 16 |
|  | Age-4 | 233 326 | 8 921 | 111 | 61 | 36 | 18 |
|  | Age-3 | 291 143 | 11 123 | 117 | 57 | 33 | 20 |
|  | Age-2 | 407 132 | 15 538 | 125 | 50 | 31 | 23 |
|  | Age-1 | 773 351 | 29 466 | 136 | 41 | 27 | 26 |
| 65-69 | Base | — | — | 39 | 64 | 34 | — |
|  | Age-5 | 99 829 | 3 819 | 61 | 49 | 25 | 8 |
|  | Age-4 | 118 861 | 4 545 | 65 | 46 | 24 | 9 |
|  | Age-3 | 138 349 | 5 288 | 68 | 45 | 24 | 10 |
|  | Age-2 | 177 332 | 6 773 | 72 | 42 | 22 | 11 |
|  | Age-1 | 294 741 | 11 240 | 76 | 39 | 21 | 13 |

^a^ The screening strategies are labeled as follows: for age-based strategies, “Age-screening frequency”; for polygenic risk-stratified strategies, “AR-10-year NPC absolute risk threshold-screening frequency”.

^b^ Early stage NPC includes those in stage I and stage II; Locoregionally advanced NPC includes those in stage III and stage IVA-B; Recurrent NPC includes those who were *de novo* metastasis and those who developed local relapse, regional relapse or metastasis after treatment.

^c^ The optimal age-based strategy was included for ease of comparison.

**Supplementary Table 14.** The results of optimal screening strategies in probabilistic sensitivity analysis

| Starting age | Screening strategy^a^ | Incremental costs^b^ | Incremental QALYs ^b^ | ICER | |
| --- | --- | --- | --- | --- | --- |
|  |  |  |  | *vs.* no screening | *vs.* preceding strategy on efficiency frontier |
| Male^c^ |  |  |  |  |  |
| 30-34 | AR-0.7%-1 | 79 257 057  (34 001 357-153 054 342) | 1 155  (565-2 013) | 71 349  (27 811-145 345) | 162 958  (67 462-356 770) |
| 35-39 | AR-0.7%-1 | 85 698 113  (36 880 333-161 403 229) | 1 204  (647-1 974) | 72 676  (28 195-149 066) | 194 279  (80 074-428 400) |
| 40-44 | AR-0.8%-1 | 65 766 999  (28 103 221-121 935 394) | 1 024  (591-1 590) | 66 181  (24 130-135 900) | 164 651  (66 489-361 702) |
| 45-49 | AR-0.7%-1 | 72 609 230  (31 971 938-131 732 137) | 945  (578-1 391) | 78 804  (30 546-159 374) | 207 315  (85 029-452 529) |
| 50-54 | AR-0.7%-1 | 56 003 262  (25 673 365-100 444 347) | 669  (436-939) | 87 181  (35 648-170 104) | 198 227  (80 949-425 476) |
| 55-59 | Age-3 | 43 953 631  (15 957 412-84 563 301) | 453  (315-606) | 98 759  (34 360-201 791) | 204 506  (78 136-428 475) |
| 60-64 | Age-4 | 21 050 787  (7 531 646-39 309 475) | 203  (149-259) | 104 615  (34 878-209 869) | 185 448  (71 296-374 245) |
| 65-69 | Age-5 | 12 008 306  (4 475 342-21 994 334) | 92  (71-113) | 131 490  (46 641-257 450) | 131 490  (46 641-257 450) |
| Female^c^ |  |  |  |  |  |
| 30-34 | AR-0.30%-1 | 37 823 463  (21 077 316-65 089 409) | 276  (132-489) | 140 915  (68 615-271 259) | 151 791  (61 073-336 286) |
| 35-39 | AR-0.30%-1 | 41 322 737  (23 009 590-70 315 334) | 309  (163-508) | 137 730  (67 695-261 396) | 147 779  (59 575-328 634) |
| 40-44 | AR-0.30%-1 | 42 130 739  (23 375 333-70 413 731) | 294  (169-456) | 146 975  (73 993-275 163) | 174 948  (72 438-391 034) |
| 45-49 | AR-0.30%-1 | 37 170 487  (20 771 691-61 193 602) | 237  (144-348) | 158 564  (81 904-291 731) | 173 479  (70 342-387 769) |
| 50-54 | AR-0.30%-1 | 29 306 829  (16 880 257-45 950 104) | 158  (102-223) | 187 044  (95 589-334 185) | 187 044  (95 589-334 185) |

^a^ The screening strategies are labeled as follows: for age-based strategies, “Age-screening frequency”; for polygenic risk-stratified strategies, “AR-10-year NPC absolute risk threshold-screening frequency”. For example, “AR-0.7%-1” indicated that individuals with a 10-year AR exceeding 0.7% undergo an EBV serological test every years.

^b^ Incremental health benefits gained, and costs incurred from age-based screening strategies relative to the no-screening strategy per 100 000 individuals with no prior NPC diagnosis, and all health utility and costs were discount at a 3% annual rate.

^c^ These results show the median and the 95% confidence interval

**
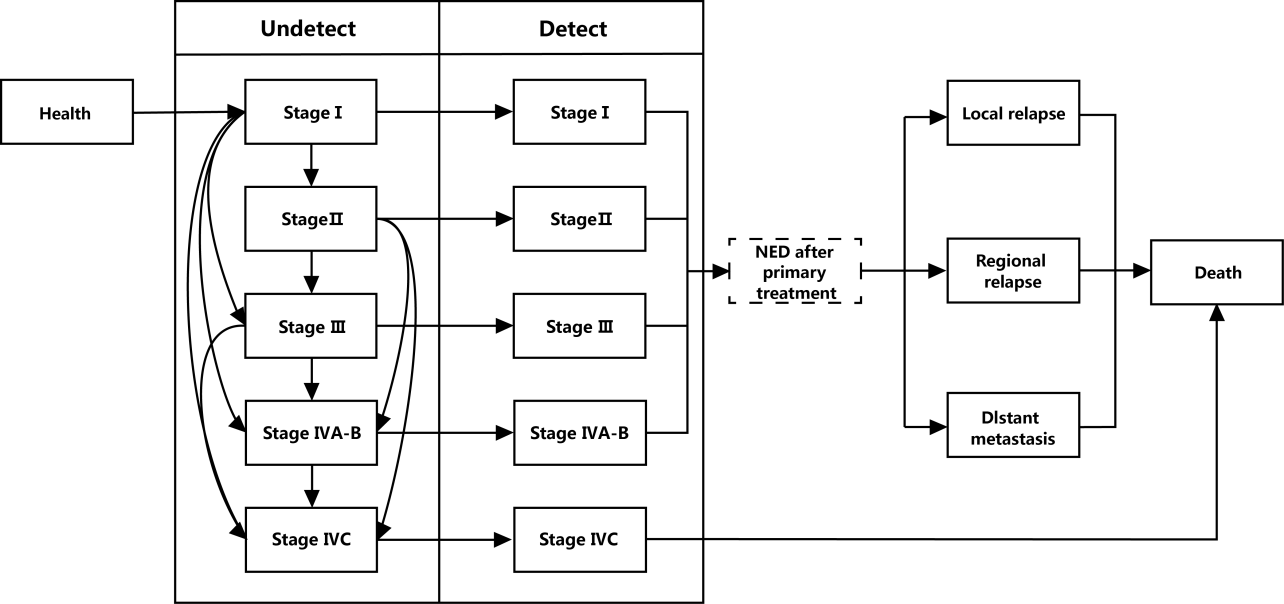
Supplementary Fig 1.** **Markov model of NPC natural history in high-risk endemic areas.** Healthy individuals could develop asymptomatic stage I NPC and progress to more advanced stages of undetected disease. The initial screening cohort comprises both healthy individuals and subjects with prevalent undiagnosed NPC. Each undetected stage of NPC could either present with symptoms or be detected by screening. Each diagnosed patient is treated with first-line radiotherapy and/or chemotherapy. Patients with each stage of non-metastatic NPC enter a state of remission, but may subsequently develop local recurrence (LR), regional recurrence (RR) and/or distant metastasis (DM), which can ultimately result in death from their disease. Patients with *de novo* metastatic NPC receive chemotherapy until dying from their disease. In each state, there is also the probability of remaining within the same state or dying from other causes.


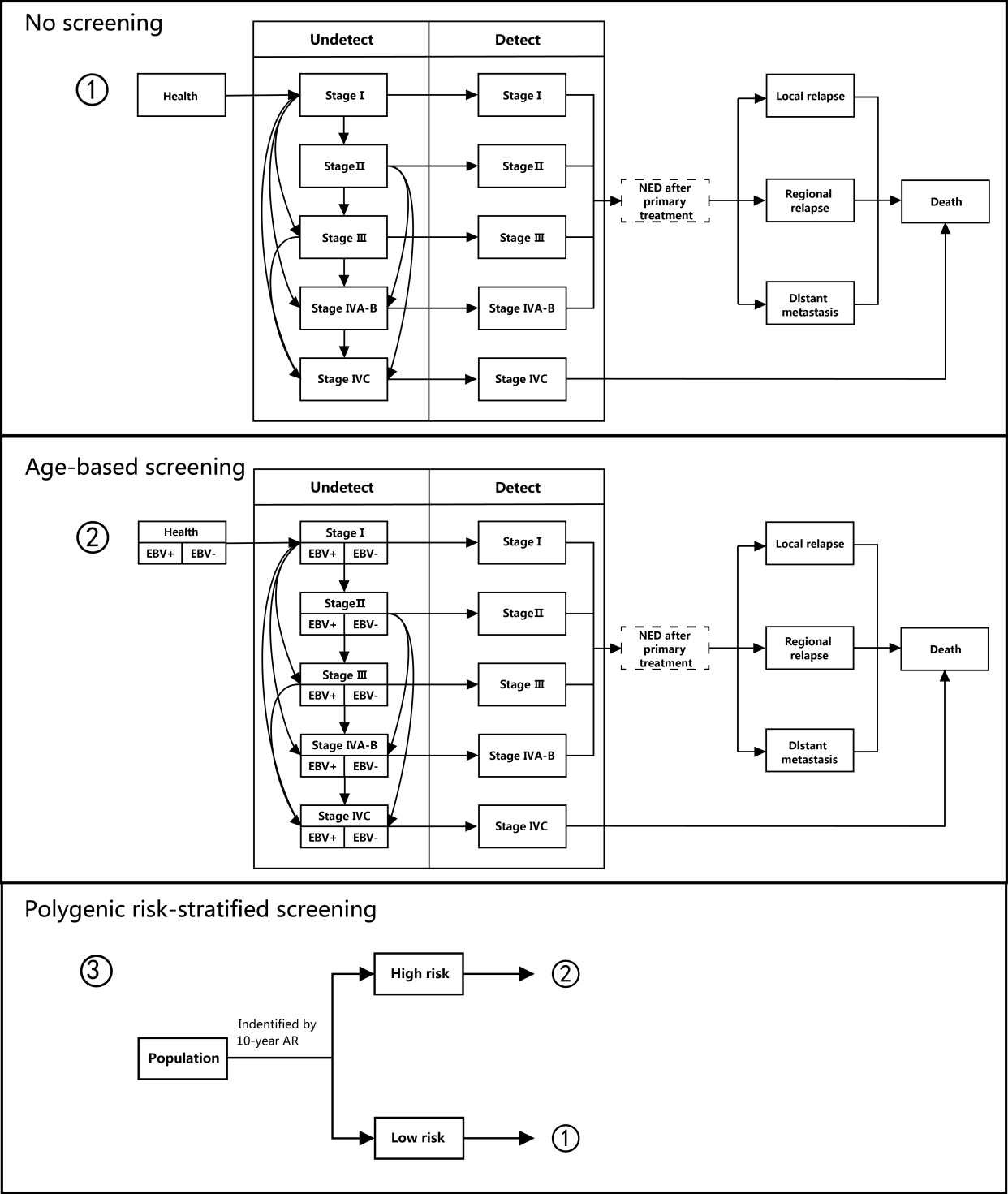


**Supplementary Fig 2. Flowchart of screening by three strategies.** No screening strategy was based on the Markov model of NPC natural history, in which individuals undergo a series of status changes, such as diagnosis due to symptoms and undergoing treatment, under natural circumstances. The age-based screening strategy optimizes the natural history model primarily by incorporating the screening process, which involves conducting EBV antibody testing on individuals in the Healthy and Undetected statuses. The transition probability from Undetected status to Detected status is determined by the sensitivity of EBV antibody testing. The polygenic risk-stratified screening strategy requires risk assessment before conducting EBV antibody testing, categorizing the population into high and low-risk groups based on a 10-year absolute risk (AR) calculated based on age, gender, and PRS. High-risk individuals undergo EBV antibody testing, while low-risk individuals are not subjected to screening.

**
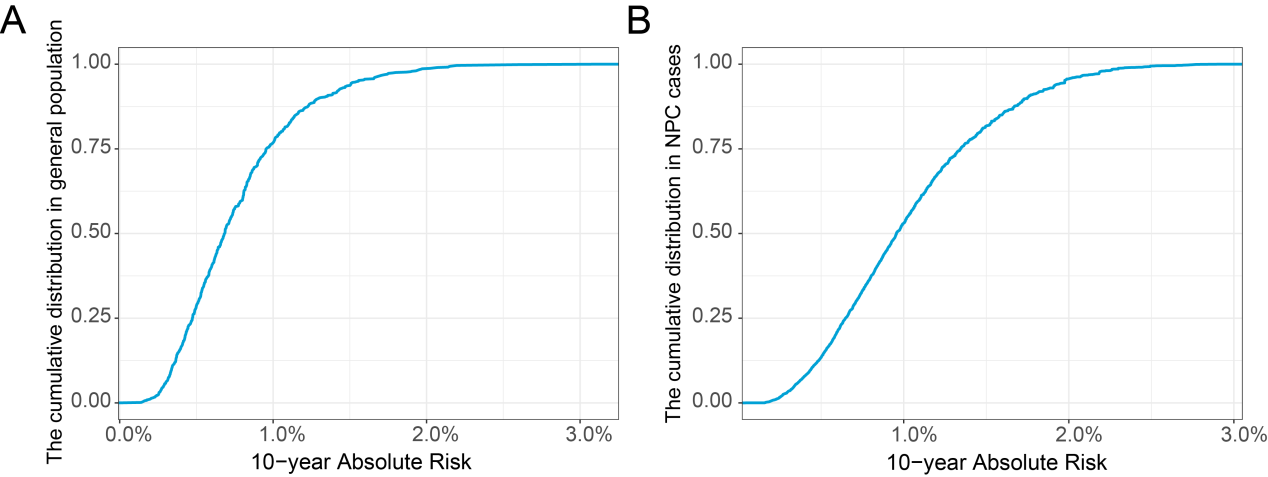
Supplementary Fig 3. The cumulative distribution of the 10-year absolute risk** (A) for general male population aged 50 years in high-risk endemic areas, and (B) for male NPC patients aged 50 years.

**
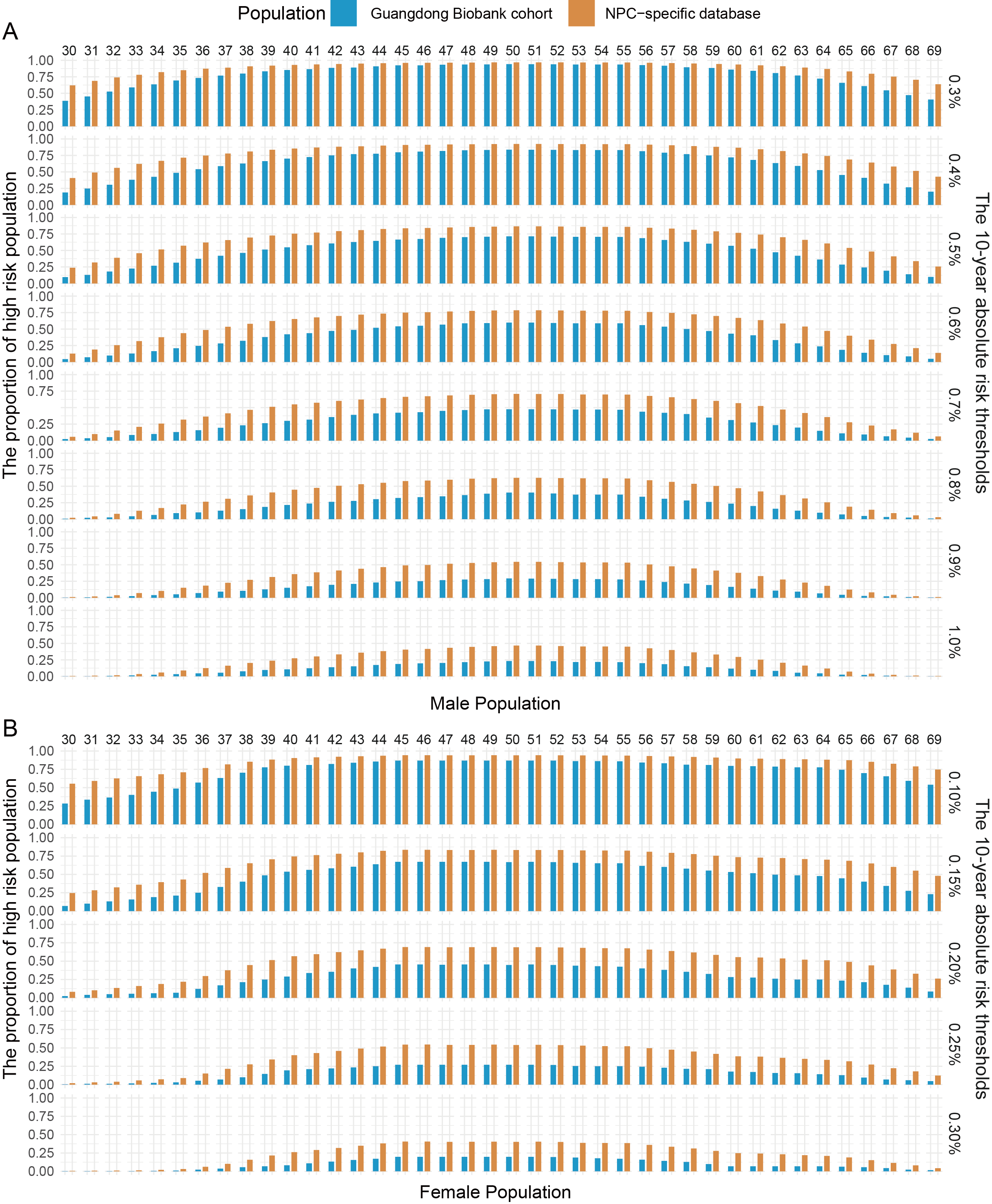
**

**Supplementary Fig 4. The proportion of high risk population in general population (blue) or NPC patients (orange) of each age group from 30 to 69 years old** (A) for the men population, and (B) for the female population.


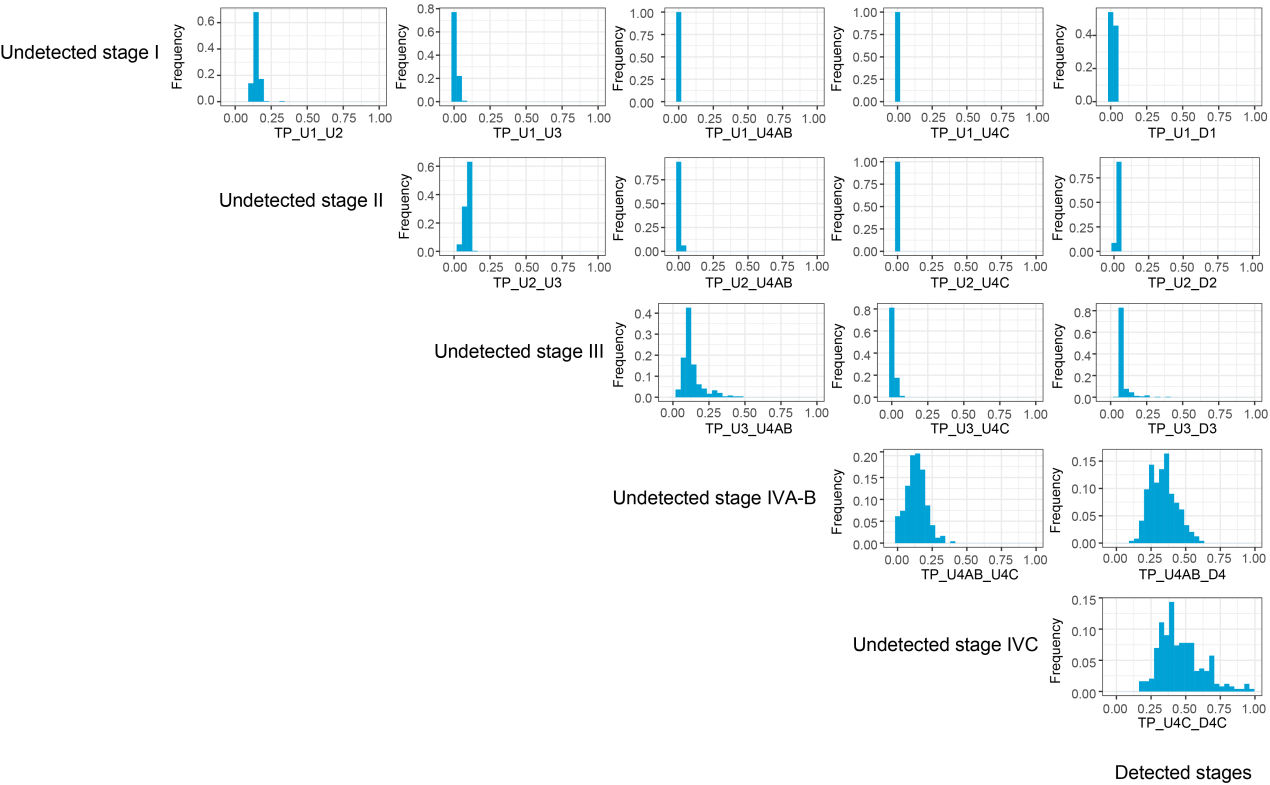


**Supplementary Fig 5.** **The transition probability distributions in the transition probability matrix between undetected states and detected states of our Markov model**. Each plot represents the distribution of transition probability that a state transition from the row state of the plot to the column state. “TP” represents transition probability, “U” represents undetected state, and “D” represents detected state. For example, TP_U3_U4AB indicates the probability of a transition from undetected stage III to undetected stage IVA-B.

**
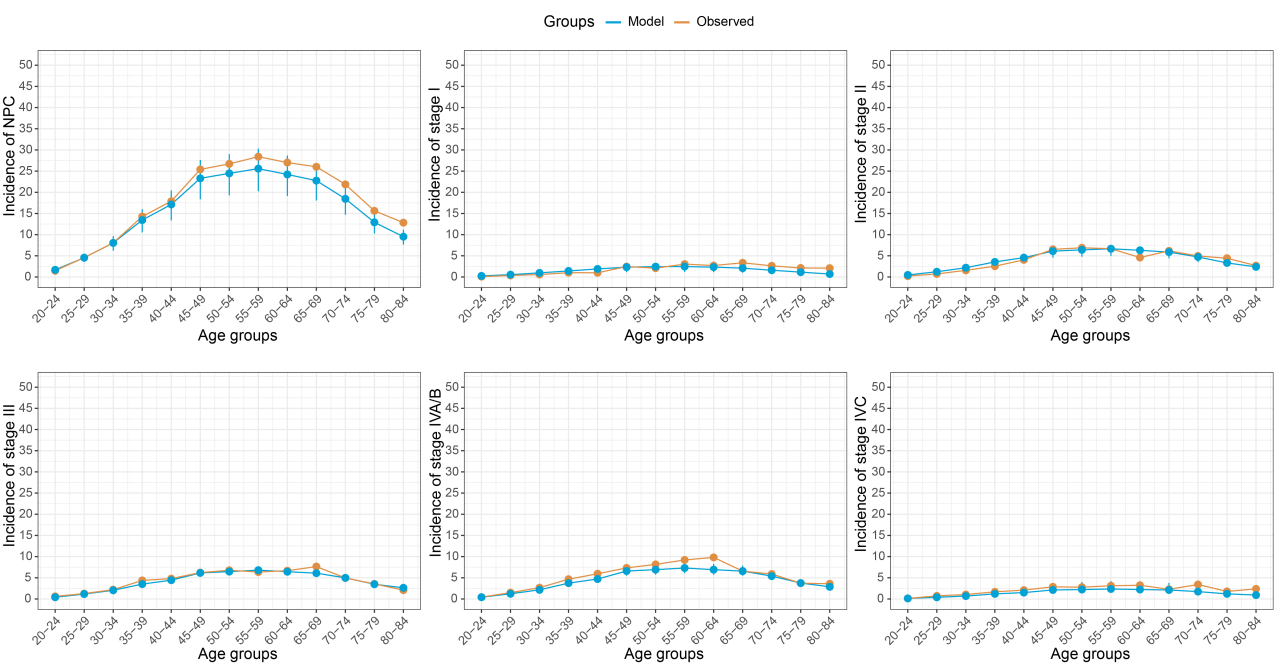
 Supplementary Fig 6. Stage- and age-specific observed incidence rate of NPC for Guangzhou registry data of CI5 (orange) and estimated incidence rate of NPC from Markov model (blue)**. The proportions of each stage in different age groups were computed by utilizing the stage-specific incidence rates from the SEER-18 database in Asian and Pacific Islander populations owing to a lack of stage-specific incidence rates in Chinese population.


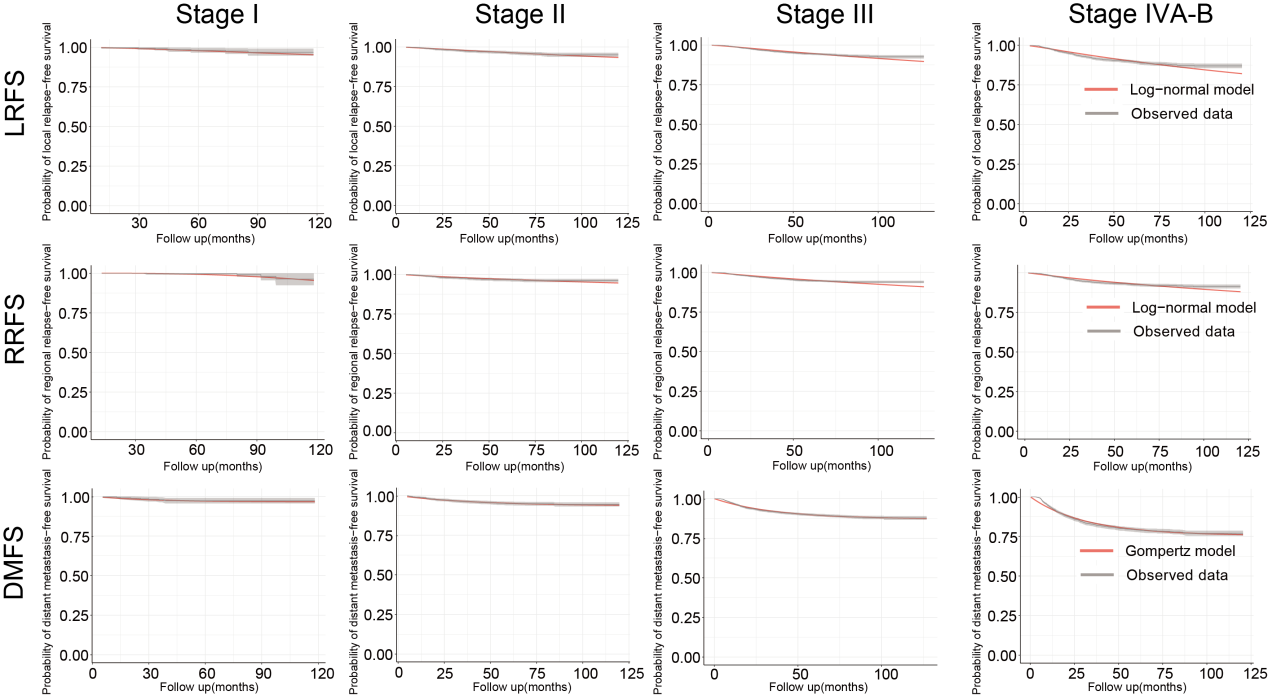


**Supplementary Fig 7. The actual and simulated stage-specific local relapse-free survival (LRFS), regional relapse-free survival (RRFS) and distant metastasis-free survival (DMFS) for non-metastatic NPC**. The figures show the performance of our Markov model to simulate disease recurrence patterns: local relapse-free survival, regional relapse-free survival and distant metastasis-free survival (red lines) compared with the observed data from real world (grey lines).


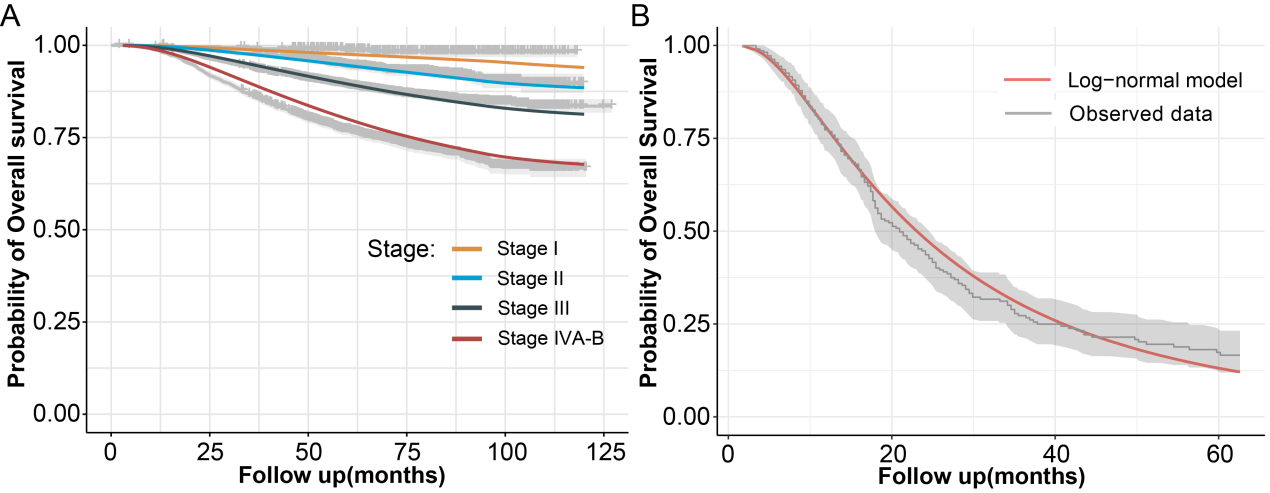


**Supplementary Fig 8. The actual and simulated stage-specific overall survival.** (A) The observed and simulated stage-specific overall survival for non-metastatic NPC. (B) The observed and simulated overall survival for metastatic NPC.


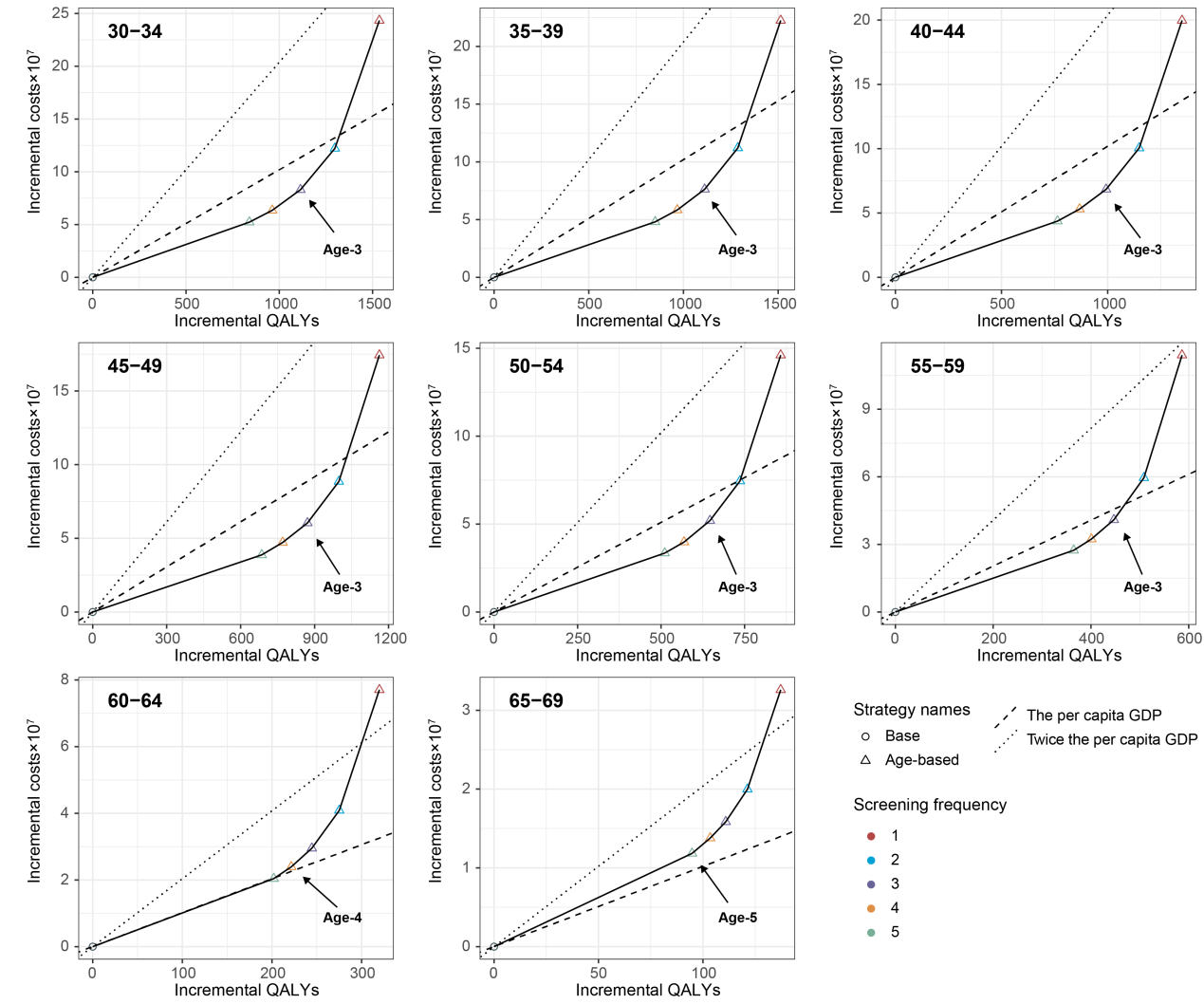


**Supplementary Fig 9. Cost-effectiveness frontiers for only age-based strategies based on the mean values of each age under the base-case analysis (100 000 male cohort members).** Incremental QALYs and incremental costs of screening strategies compared with the no intervention scenario.


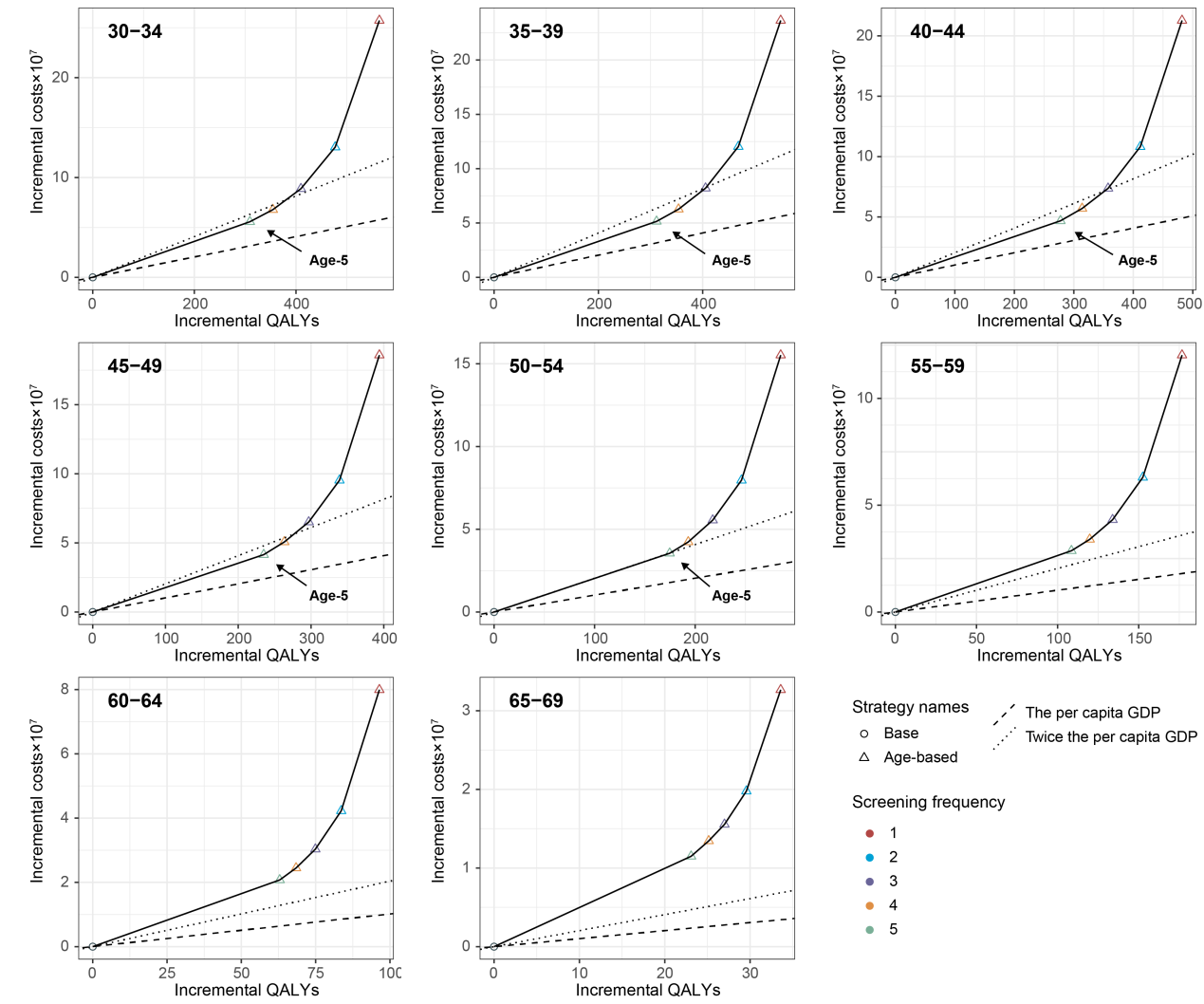


**Supplementary Fig 10. Cost-effectiveness frontiers for only age-based strategies based on the mean values of each age under the base-case analysis (100 000 female cohort members).** Incremental QALYs and incremental costs of screening strategies compared with the no intervention scenario

**
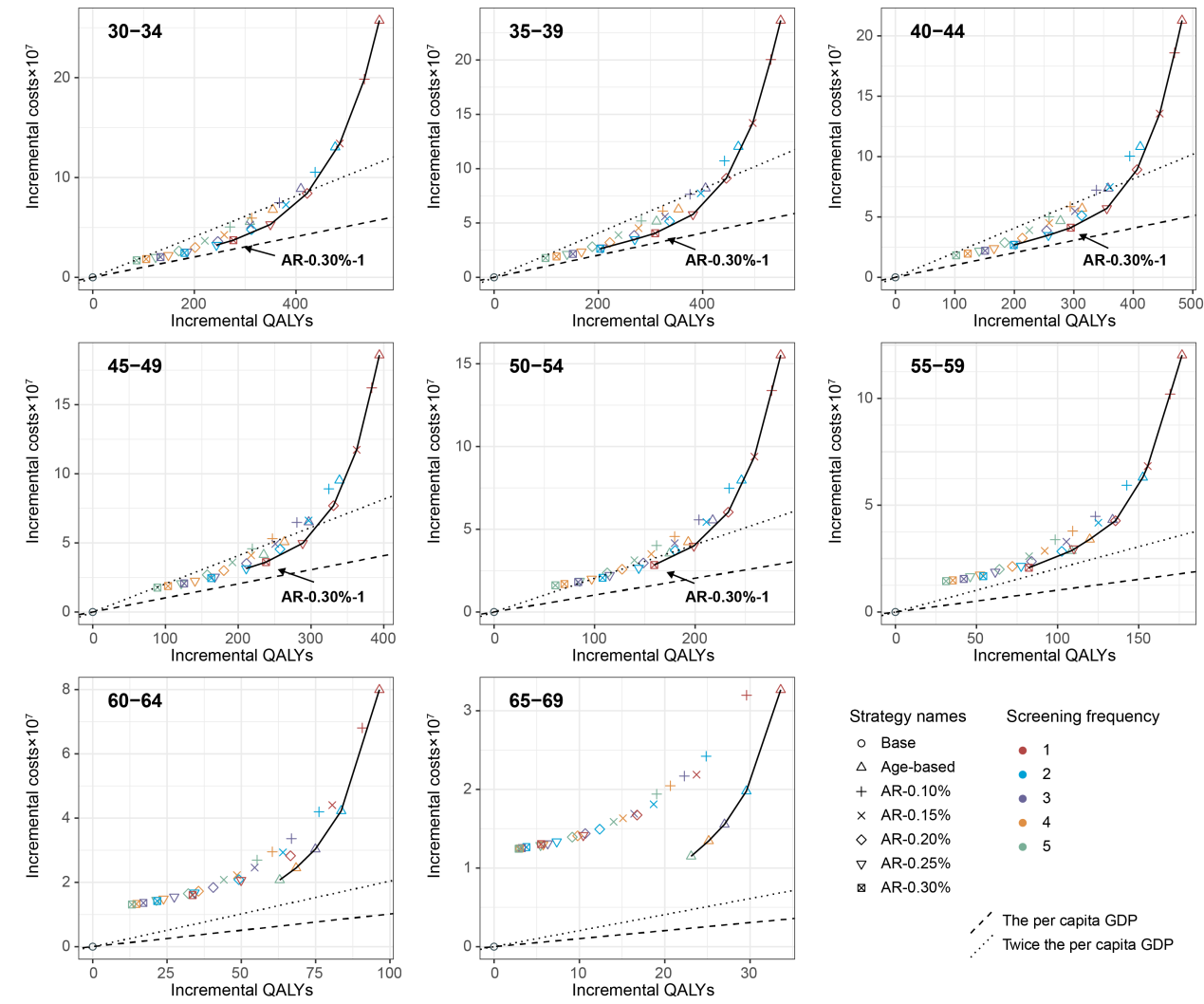
**

**Supplementary Fig 11. Cost-effectiveness frontiers for all screening strategies based on the mean values of each age under the base-case analysis (100 000 female cohort members).** Incremental QALYs and incremental costs of screening strategies compared with the no intervention scenario. The line on the each plot was cost-effectiveness efficiency frontier. The strategies on the upper left of the frontier are dominated by the strategies on the lower right of them.

**
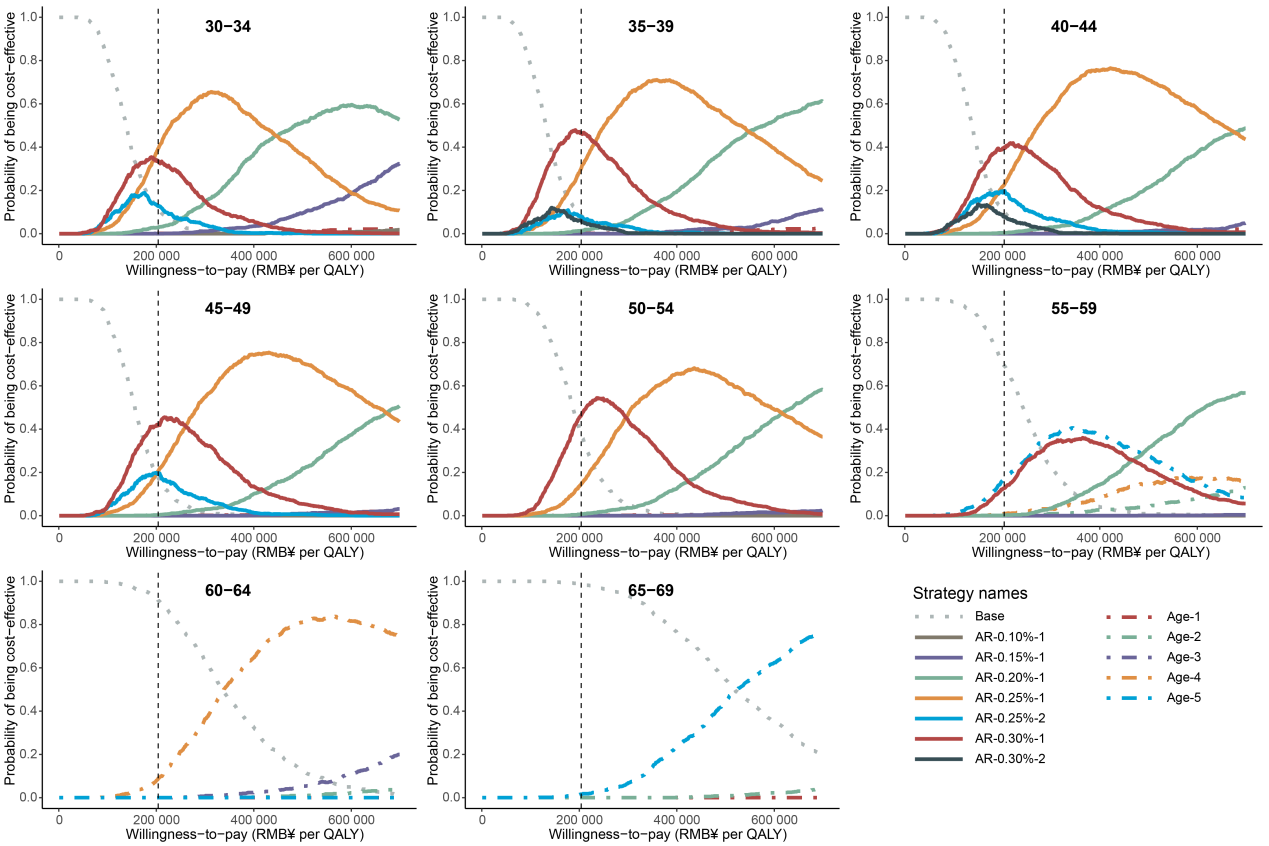
**

**Supplementary Fig 12. Cost-effectiveness acceptability curves for all strategies on the cost-effectiveness efficiency frontier in female population.** The grey dotted line represents the curves of no screening scenario, the dot dash lines represent the curves of age-based strategies, and the solid lines represent the curves of polygenic risk-stratified strategies. The vertical grey dash line represents the WTP of twice per capita GDP. The screening strategies are labeled as follows: for age-based strategies, Age-screening frequency; for polygenic risk-stratified strategies, AR-10-year NPC absolute risk threshold-screening frequency.

**
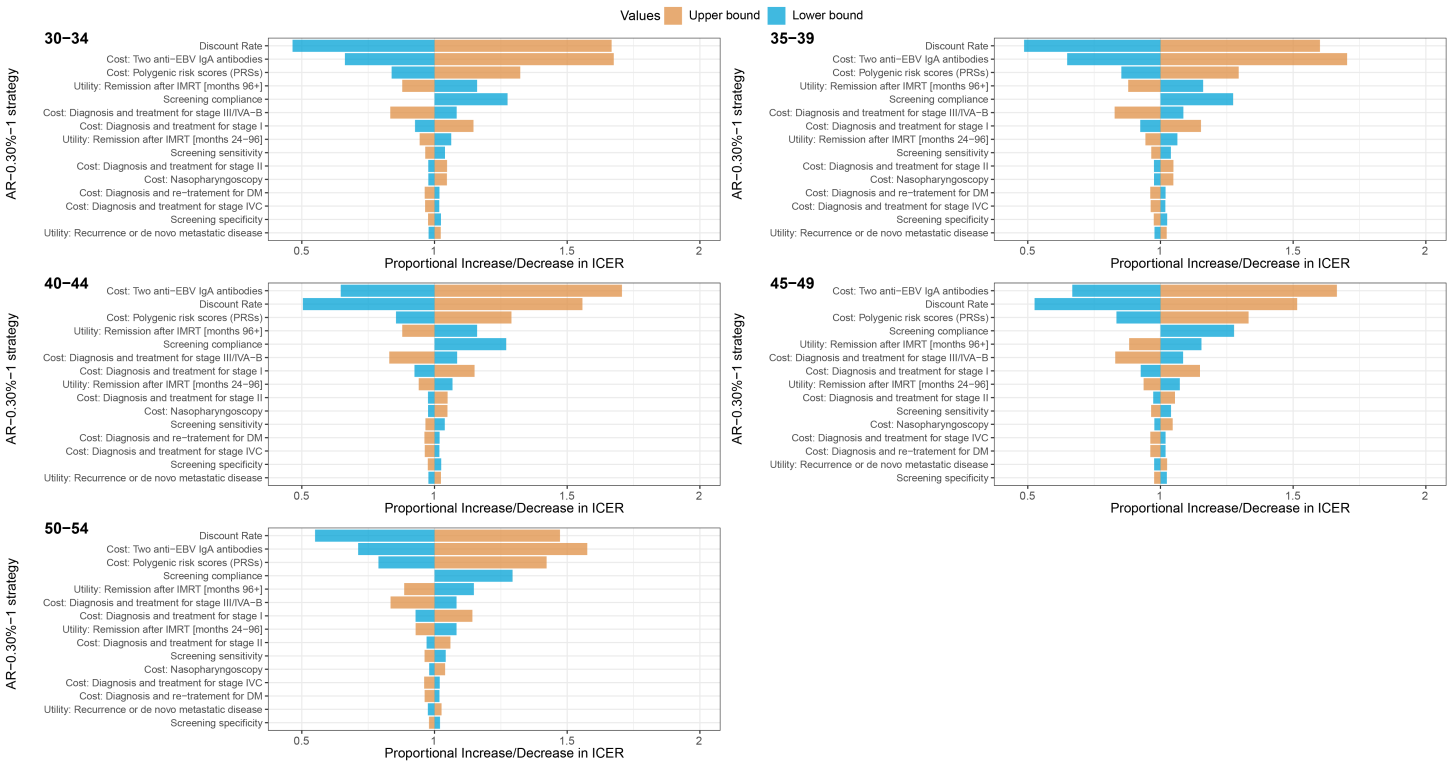
 Supplementary Fig 13. Tornado plots for one-way deterministic sensitivity analysis of the optimal strategies compared with the no screening strategy in female population.** Compared to no screening strategy, a proportional increase or decrease in incremental cost-effectiveness ratio is plotted for upper (orange) and lower (blue) bounds of model parameters. The optimal strategies were polygenic risk-stratified strategies for 30-34, 35-39, 40-44, 45-49 and 50-54 age groups. The tornado plots are not shown for 55-59, 60-64 and 65-69 age groups as no optimal screening strategies were identified for these age groups.

**Reference**

1. Miller JA, Le QT, Pinsky BA, Wang H. Cost-Effectiveness of Nasopharyngeal Carcinoma Screening With Epstein-Barr Virus Polymerase Chain Reaction or Serology in High-Incidence Populations Worldwide. *Journal of the National Cancer Institute* 2021; **113**(7): 852-62.

2. World Health Organization, Life tables by country, China. 2019. https://apps.who.int/gho/data/view.main.60340. Accessed 2022.

3. Bray F, Colombet M, Mery L, et al. Cancer Incidence in Five Continents, Vol. XI (electronic version). Lyon: International Agency for Research on Cancer. Available from: https://ci5.iarc.fr, accessed 2022.

4. Nash JC, Varadhan R. Unifying Optimization Algorithms to Aid Software System Users: optimx for R. *Journal of Statistical Software* 2011; **43**(9): 1 - 14.

5. Lv JW, Chen YP, Huang XD, et al. Hepatitis B virus screening and reactivation and management of patients with nasopharyngeal carcinoma: A large-scale, big-data intelligence platform-based analysis from an endemic area. *Cancer* 2017; **123**(18): 3540-9.

6. Latimer NR. Survival analysis for economic evaluations alongside clinical trials--extrapolation with patient-level data: inconsistencies, limitations, and a practical guide. *Medical decision making : an international journal of the Society for Medical Decision Making* 2013; **33**(6): 743-54.

7. Wan X, Zhang Y, Tan C, Zeng X, Peng L. First-line Nivolumab Plus Ipilimumab vs Sunitinib for Metastatic Renal Cell Carcinoma: A Cost-effectiveness Analysis. *JAMA oncology* 2019; **5**(4): 491-6.

8. Li WZ, Lv SH, Liu GY, et al. Development of a Prognostic Model to Identify the Suitable Definitive Radiation Therapy Candidates in de Novo Metastatic Nasopharyngeal Carcinoma: A Real-World Study. *International journal of radiation oncology, biology, physics* 2021; **109**(1): 120-30.

9. Guyot P, Ades AE, Ouwens MJ, Welton NJ. Enhanced secondary analysis of survival data: reconstructing the data from published Kaplan-Meier survival curves. *BMC medical research methodology* 2012; **12**: 9.

10. He YQ, Wang TM, Ji M, et al. A polygenic risk score for nasopharyngeal carcinoma shows potential for risk stratification and personalized screening. *Nature communications* 2022; **13**(1): 1966.

11. Wu CF, Lin L, Mao YP, et al. Liquid biopsy posttreatment surveillance in endemic nasopharyngeal carcinoma: a cost-effective strategy to integrate circulating cell-free Epstein-Barr virus DNA. *BMC medicine* 2021; **19**(1): 193.

12. Tang LL, Chen YP, Chen CB, et al. The Chinese Society of Clinical Oncology (CSCO) clinical guidelines for the diagnosis and treatment of nasopharyngeal carcinoma. *Cancer communications* 2021; **41**(11): 1195-227.

13. Lee N, Harris J, Garden AS, et al. Intensity-modulated radiation therapy with or without chemotherapy for nasopharyngeal carcinoma: radiation therapy oncology group phase II trial 0225. *Journal of clinical oncology : official journal of the American Society of Clinical Oncology* 2009; **27**(22): 3684-90.

14. Lee NY, Zhang Q, Pfister DG, et al. Addition of bevacizumab to standard chemoradiation for locoregionally advanced nasopharyngeal carcinoma (RTOG 0615): a phase 2 multi-institutional trial. *The Lancet Oncology* 2012; **13**(2): 172-80.

15. Zhou GQ, Wu CF, Zhang J, et al. Cost-Effectiveness Analysis of Routine Magnetic Resonance Imaging in the Follow-Up of Patients With Nasopharyngeal Carcinoma After Intensity Modulated Radiation Therapy. *International journal of radiation oncology, biology, physics* 2018; **102**(4): 1382-91.

16. Ji MF, Sheng W, Cheng WM, et al. Incidence and mortality of nasopharyngeal carcinoma: interim analysis of a cluster randomized controlled screening trial (PRO-NPC-001) in southern China. *Annals of oncology : official journal of the European Society for Medical Oncology* 2019; **30**(10): 1630-7.

17. Yang J, Han J, He J, et al. Real-World Cost-Effectiveness Analysis of Gemcitabine and Cisplatin Compared to Docetaxel and Cisplatin Plus Fluorouracil Induction Chemotherapy in Locoregionally Advanced Nasopharyngeal Carcinoma. *Frontiers in oncology* 2020; **10**: 594756.

18. Liu YP, Lv X, Zou X, et al. Minimally invasive surgery alone compared with intensity-modulated radiotherapy for primary stage I nasopharyngeal carcinoma. *Cancer communications* 2019; **39**(1): 75.

19. Zhou GQ, Wu CF, Deng B, et al. An optimal posttreatment surveillance strategy for cancer survivors based on an individualized risk-based approach. *Nature communications* 2020; **11**(1): 3872.

20. Miller JA, Sahoo MK, Yamamoto F, et al. Multiplex Epstein-Barr virus BALF2 genotyping detects high-risk variants in plasma for population screening of nasopharyngeal carcinoma. *Molecular cancer* 2022; **21**(1): 154.

21. Han F, Zhao C, Huang SM, et al. Long-term outcomes and prognostic factors of re-irradiation for locally recurrent nasopharyngeal carcinoma using intensity-modulated radiotherapy. *Clinical oncology* 2012; **24**(8): 569-76.

22. Tian YM, Tian YH, Zeng L, et al. Prognostic model for survival of local recurrent nasopharyngeal carcinoma with intensity-modulated radiotherapy. *British journal of cancer* 2014; **110**(2): 297-303.

23. Tian YM, Huang WZ, Yuan X, Bai L, Zhao C, Han F. The challenge in treating locally recurrent T3-4 nasopharyngeal carcinoma: the survival benefit and severe late toxicities of re-irradiation with intensity-modulated radiotherapy. *Oncotarget* 2017; **8**(26): 43450-7.

24. Lo WC, Wang CP, Ko JY, et al. Salvage treatment for isolated regional failure of nasopharyngeal carcinoma after primary radiotherapy. *Annals of surgical oncology* 2012; **19**(3): 1001-8.

25. Li X, Lin C, Yan J, et al. Establishment of a prognostic scoring model for regional recurrent nasopharyngeal carcinoma after neck dissection. *Cancer biology & medicine* 2020; **17**(1): 227-36.

26. Zheng WH, He XJ, Chen FP, et al. Establishing M1 stage subdivisions by incorporating radiological features and Epstein-Barr virus DNA for metastatic nasopharyngeal carcinoma. *Annals of translational medicine* 2020; **8**(4): 83.

27. Shen L, Li W, Wang S, et al. Image-based Multilevel Subdivision of M1 Category in TNM Staging System for Metastatic Nasopharyngeal Carcinoma. *Radiology* 2016; **280**(3): 805-14.
